# Probiotic-Postbiotic Synergy and Microbial Co-Culture Strategies for Hyperuricemia: Gut Microbiota and Functional Food Perspectives

**DOI:** 10.3390/foods15203580

**Published:** 2026-10-09

**Authors:** Alsurabi Khawlah, Xiangfeng Zheng, Yunzhe Jia, Zhirong Wang, Zhenquan Yang

**Affiliations:** College of Food Science and Engineering, Yangzhou University, Yangzhou 225009, China

**Keywords:** probiotics, synbiotics, hyperuricemia, gut microbiota, purine metabolism, uric acid transport, functional foods

## Abstract

Hyperuricemia is a common metabolic disorder associated with gout, chronic kidney disease, and cardiovascular complications. Despite the availability of urate-lowering therapies such as xanthine oxidase inhibitors, their long-term use may be constrained in some patients due to adverse effects, contraindications, or suboptimal adherence. In this context, probiotics have emerged as promising functional food components with potential roles in metabolic regulation and disease prevention. This review comprehensively examines synergistic strategies for uric acid reduction, focusing on the combined use of probiotics, prebiotics, synbiotics, and postbiotics. Special attention is given to emerging approaches, such as microbial co-culture systems, including yeast–lactic acid bacteria combinations, as innovative sources of bioactive metabolites with enhanced functional potential. Preclinical evidence suggests that probiotic-based interventions may influence uric acid homeostasis through several interconnected pathways, including modulation of gut microbiota composition, purine metabolism and xanthine oxidase activity, regulation of urate transporters such as URAT1 and ABCG2, and attenuation of oxidative stress and inflammatory signaling. Additionally, experimental studies indicate that synergistic formulations may support intestinal barrier function and metabolic cross-feeding, particularly through postbiotic and co-culture-derived metabolites. However, most of the available evidence remains preclinical, while human clinical studies are still limited, heterogeneous in design, and insufficient to establish comparative therapeutic efficacy. Accordingly, probiotic-, postbiotic-, and co-culture-based approaches should currently be regarded as emerging adjunctive strategies with potential multitarget effects rather than alternatives to established urate-lowering therapies. Future studies should include well-designed clinical trials, mechanistic studies in human models, and the development of personalized and next-generation probiotic formulations to support clinical translation.

## 1. Introduction

### 1.1. Global Burden of Hyperuricemia

In recent decades, hyperuricemia, or serum uric acid concentrations above normal values, has become a significant public health problem worldwide. Its prevalence has increased in many populations, with lifestyle-related factors, such as excessive consumption of fructose- and purine-rich foods and physical inactivity, contributing to this growing burden [1,2]. Epidemiological studies indicate that hyperuricemia affects a substantial proportion of the adult population, although its prevalence varies according to geographic region, population characteristics, sex, age, and the diagnostic threshold used [1,3]. For example, a large population-based study involving 30,158 adults in the Beijing–Tianjin–Hebei region of China reported an overall hyperuricemia prevalence of 19.37% [2].

The increasing prevalence of both hyperuricemia and gout contributes to a growing burden on healthcare systems [1,3]. Importantly, hyperuricemia has also been associated with several chronic metabolic, renal, and cardiovascular disorders, indicating that its clinical relevance extends beyond its role as a risk factor for gout [2]. It has been suggested that there is a connection between hyperuricemia and metabolic syndrome, which includes dyslipidemia, obesity, insulin resistance, and hypertension [2,4]. In addition, decreased renal function decreases uric acid excretion, which, in turn, leads to a vicious cycle of uric acid accumulation; conversely, persistent hyperuricemia has been associated with mechanisms that may contribute to renal injury and chronic kidney disease progression [2,4]. Hyperuricemia can also be associated with cardiovascular diseases, including hypertension, coronary artery disease, and heart failure, through mechanisms involving endothelial dysfunction, oxidative stress, and inflammation [2,4]. However, these epidemiological associations should not uniformly be interpreted as evidence of direct causality [2]. Taken together, these results highlight the systemic nature of hyperuricemia and the urgent need for effective and sustainable hyperuricemia management strategies [1,2].

### 1.2. Limitations of Current Therapeutic Strategies

When pharmacological urate-lowering therapy is indicated, conventional treatment primarily involves reducing urate production or enhancing urate excretion [2,5]. Uricosuric agents (e.g., benzbromarone) promote urate excretion from the kidneys, whereas xanthine oxidase inhibitors (e.g., allopurinol and febuxostat) are used to reduce uric acid production [2,4]. These therapies can effectively reduce serum uric acid levels; however, treatment selection and long-term use require consideration of the clinical indication, comorbidities, potential adverse effects, and individual patient factors [4]. Allopurinol is one of the most commonly used medications and is associated with rare but severe hypersensitivity reactions, including Stevens–Johnson syndrome [6]. Febuxostat is also an effective xanthine oxidase inhibitor; however, its cardiovascular safety has been debated, and the available evidence does not indicate a uniform increase in cardiovascular risk across all patient populations [5]. Benzbromarone is an effective uricosuric agent, although its use has been restricted in some regions because of concerns regarding hepatotoxicity [5].

Beyond drug-related safety considerations, long-term management can also be influenced by patients’ perceptions of treatment necessity and their willingness to initiate or continue urate-lowering therapy [7]. In individuals with asymptomatic hyperuricemia, recent evidence indicates considerable variability in perceived need for pharmacological treatment and concerns regarding long-term medication use [7]. Importantly, the decision to initiate pharmacological urate-lowering therapy in asymptomatic hyperuricemia remains clinically context-dependent, and recommendations are not uniform across clinical settings [5,7]. In addition, a pharmacological approach typically targets one pathway (e.g., increasing uric acid excretion or blocking its synthesis), without cosdering the multifactorial nature of the disease. These limitations highlight the need for additional or alternative approaches that are effective, safe, and sustainable, and which can address multiple processes simultaneously [7].

### 1.3. Functional Foods as Complementary Strategies

Functional foods have gained increasing interest as complementary dietary strategies for metabolic disorders, including hyperuricemia. Foods with health benefits in addition to their nutritional value are typically categorized as functional foods and often contain bioactive compounds that influence physiological functions [8]. In the context of hyperuricemia, these foods should currently be considered complementary or adjunctive strategies rather than replacements for established urate-lowering therapy. Regulatory guidelines for functional foods vary between regions but tend to focus significantly on efficacy, safety, and scientific substantiation of health claims in the product associated with individual products [9]. One of the most studied components of functional foods is probiotics. Probiotics are live microorganisms that, when administered in adequate amounts, confer a health benefit on the host [9,10]. Probiotics have a unique role as they can influence the gut microbiota and host–microbe interactions that contribute to metabolic homeostasis [9,10]. For conceptual clarity, probiotics should be distinguished from prebiotics, synbiotics, and postbiotics. Prebiotics are substrates selectively utilized by host microorganisms that confer a health benefit [11], whereas synbiotics comprise live microorganisms together with substrate(s) selectively utilized by host microorganisms to confer a health benefit [12]. Postbiotics are preparations of inanimate microorganisms and/or their components that confer a health benefit on the host [13]. Accordingly, isolated microbial metabolites should not automatically be considered postbiotics unless they form part of a preparation that meets the accepted postbiotic definition [13,14].

There is evidence that the gut microbiota participates in purine metabolism and may influence uric acid production and excretion [11]. Probiotics have been shown to enhance gut barrier function, modulate immune responses, and influence microbial composition. However, direct evidence linking all of these effects to urate lowering remains predominantly preclinical, and mechanistic findings from other metabolic or inflammatory models should not be interpreted as direct evidence in human hyperuricemia [14]. In recent years, research has gone beyond the use of probiotics and now involves synbiotic approaches, postbiotic preparations, and fermentation-derived microbial products. These approaches may offer practical advantages related to formulation stability, standardization, and delivery, although their efficacy and safety depend on the specific preparation and intended application [13,14]. Diet-derived bioactive compounds provide an additional link between functional foods and the gut microbiota. Polyphenol-rich foods, including those characteristic of Mediterranean dietary patterns, can influence microbial composition, while gut microorganisms can biotransform poorly absorbed polyphenols into metabolites with distinct biological activities [15,16]. These interactions provide mechanistic support for microbiota-directed nutritional strategies, although they should not be interpreted as direct evidence of urate-lowering efficacy [15,17]. In particular, microbial co-culture systems involving compatible yeasts and lactic acid bacteria have emerged as a strategy to modify fermentation performance and expand the diversity of fermentation-derived metabolites. Interactions between yeasts and lactic acid bacteria can involve metabolic cross-feeding, nutrient exchange, and modification of the fermentation environment [14,18]. These metabolites, such as fatty acids, peptides, and exopolysaccharides, may have potential functional effects relevant to gut and metabolic health. However, direct evidence demonstrating clinically meaningful urate-lowering effects of yeast–LAB co-culture systems remains limited; therefore, these systems should currently be considered emerging experimental and functional food platforms rather than established therapeutic strategies for hyperuricemia [18].

### 1.4. Scope and Objectives

Given the multifactorial nature of hyperuricemia and the limitations that may affect long-term management in some patients, there is growing interest in complementary approaches that can influence multiple biological pathways involved in urate homeostasis [2,5]. The concept of synergy in probiotic-based interventions is the focus of this review, which includes probiotics, prebiotics, synbiotics, postbiotics, and bioactive compounds [15,19]. This review addresses the complex mechanisms involved in the metabolism of uric acid and the influence of different microbiota-directed and food-derived interventions on it. Particular attention is paid to the gut–kidney–liver axis, microbial control of purine metabolism, uric acid transporter modulation, and anti-inflammatory and antioxidant effects of microbial metabolites [15]. Importantly, the evidence discussed throughout this review is interpreted according to its level of support: direct evidence obtained from hyperuricemia models or human hyperuricemia studies is distinguished from indirect evidence derived from related metabolic, renal, inflammatory, or microbiome models and from mechanistic hypotheses that require further validation. In addition, this review examines emerging microbial co-culture systems involving compatible yeasts and lactic acid bacteria as potential functional-fermentation platforms. Such systems may support metabolic cross-feeding and generate diverse fermentation-derived products, although their direct anti-hyperuricemic efficacy remains insufficiently established [14,18]. Several previous reviews have examined probiotics, dietary bioactive compounds, dietary patterns, plant-derived nutraceuticals, and food-derived peptides in relation to hyperuricemia [19,20,21,22]. The present review differs in its integrative emphasis on probiotic–postbiotic relationships, synergistic formulations, yeast–LAB co-culture as an emerging fermentation platform, and the explicit distinction between direct hyperuricemia evidence and indirect mechanistic evidence. This framework is intended to connect mechanistic microbiome research with functional food development while avoiding interpretation of emerging approaches as established clinical treatments. The primary goals are to evaluate the existing data, determine critical mechanistic pathways, and critically synthesize the available evidence within a structured narrative framework. Further, this study aims to uncover gaps in knowledge and propose future directions, particularly in clinical translation, personalized nutrition, and next-generation probiotic technologies.

## 2. The Gut–Kidney–Liver Axis in Uric Acid Homeostasis

**Figure 1:**
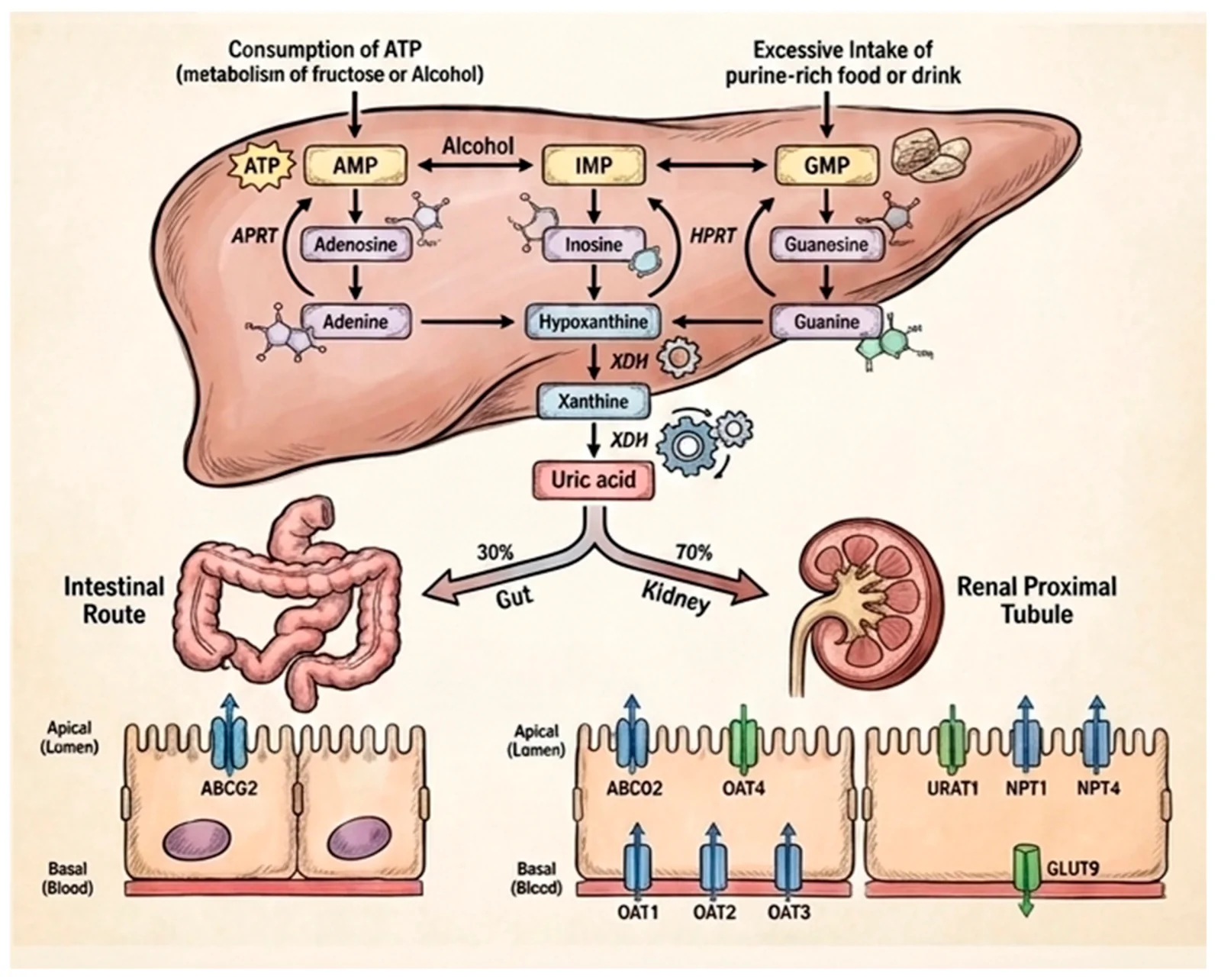
Mechanisms of hyperuricemia. Schematic overview of purine metabolism, uric acid production, and intestinal and renal urate handling. Purine nucleotides are metabolized through hypoxanthine and xanthine to uric acid via xanthine dehydrogenase/oxidase (XDH/XO). Approximately 70% of urate elimination occurs through the renal route and about 30% through the gastrointestinal route. In the kidney, most filtered urate is reabsorbed, with URAT1 and GLUT9 contributing importantly to tubular urate reabsorption. The lower panel illustrates the principal intestinal and renal transport pathways. Blue and green transporter symbols distinguish different urate transport processes, while arrows indicate the direction of metabolic conversion or urate transport. Percentage values indicate approximate physiological contributions to urate elimination. Colors are used for visual distinction and do not represent quantitative differences unless otherwise indicated [23].

The liver, kidneys, and gastrointestinal tract are intimately involved in uric acid homeostasis, a complex multiorgan process. This integrated system, also known as the gut–kidney–liver axis, is crucial for maintaining serum urate within physiological limits [23,24,25]. This axis can be disturbed at any level, leading to hyperuricemia and impaired uric acid metabolism [23,24,25]. There is growing evidence that gut bacteria and their metabolites may influence this axis, in addition to host metabolic pathways [15,26]. However, several microbiota-related mechanisms remain supported mainly by preclinical or mechanistic evidence and require further validation in human hyperuricemia.The coordinated roles of the liver, kidneys, and gastrointestinal tract in uric acid production, transport, and elimination are summarized in Figure 1 [23].

### 2.1. Physiological Overview of Uric Acid Metabolism

**Figure 2:**
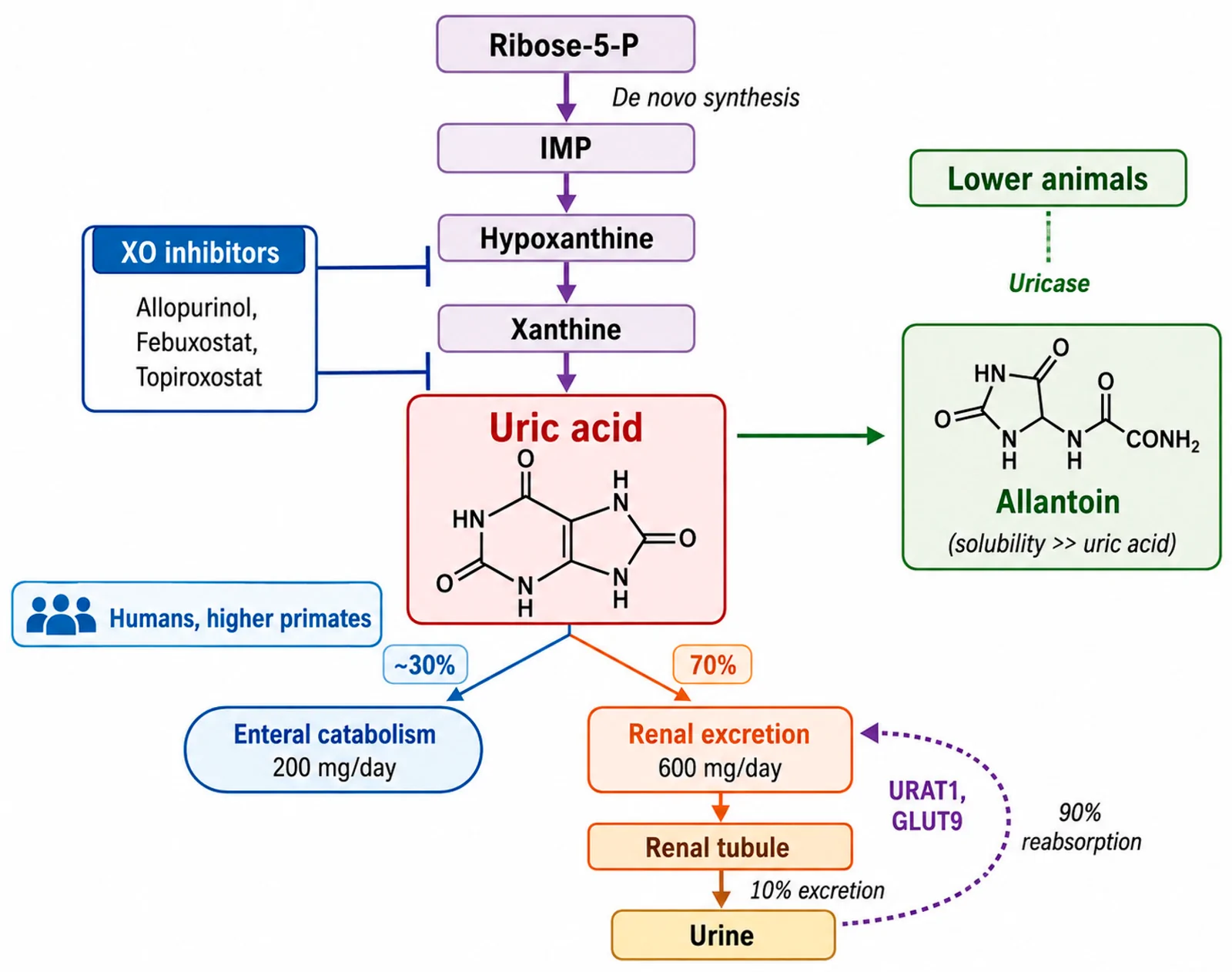
Schematic representation of uric acid synthesis and metabolism. Purine degradation proceeds from IMP through hypoxanthine and xanthine to uric acid. Xanthine oxidase (XO) inhibitors, including allopurinol, febuxostat, and topiroxostat, inhibit the oxidative steps that lead to uric acid formation. In humans and higher primates, uric acid is the end product of purine metabolism, whereas in lower animals, uricase converts uric acid to the more soluble allantoin. The schematic further illustrates the approximate distribution of urate elimination between the intestinal (~30%) and renal (~70%) routes. In the kidney, URAT1 and GLUT9 are shown as major transporters involved in urate reabsorption, with approximately 10% ultimately excreted in the urine. Blue elements indicate the human/higher-primate and intestinal pathways, orange elements indicate renal handling and urinary excretion, green elements indicate uricase-mediated conversion to allantoin in lower animals, and the purple dashed arrow indicates renal urate reabsorption through URAT1 and GLUT9. Solid arrows indicate the direction of metabolic conversion or excretion, whereas inhibitory bars indicate the sites of action of XO inhibitors. The numerical percentages represent the approximate physiological proportions indicated in the schematic.

The final product of purine metabolism in humans is uric acid, which cannot be metabolized to the more soluble allantoin by humans and higher primates. Purine nucleotide synthesis occurs through two principal pathways in purine metabolism: the salvage pathway and de novo synthesis. The de novo synthesis pathway generates purine nucleotides starting from small molecular building blocks such as CO_2_, amino acids, and tetrahydrofolate derivatives [27]. This pathway is tightly regulated, although increased purine turnover can increase the amount of purine available for degradation to uric acid [27,28]. In the salvage pathway, on the other hand, free purine bases such as hypoxanthine and guanine are recycled into nucleotides, thereby limiting their degradation to uric acid.. Dysregulation of these processes may contribute to increased uric acid levels through de novo synthesis or reduced salvage activity [28]. Hypoxanthine is converted to xanthine and then to uric acid by xanthine oxidase, which is responsible for the final steps in purine metabolism. This enzyme is a key therapeutic target and a major cause of hyperuricemia [24]. Renal excretion is the main method of eliminating uric acid, accounting for approximately two-thirds of total urate elimination [24,25]. Filtration, reabsorption, secretion, and post-secretory reabsorption in the proximal tubules are all part of the tightly controlled renal management of uric acid. A number of transport proteins are essential for this process. Major reabsorptive transporters that aid in the absorption of urate from the tubular lumen back into the circulation are URAT1 and GLUT9. Conversely, transporters such as ABCG2 and ABCC2 increase the excretion of urate by promoting its secretion into the tubular lumen. Serum uric acid levels can be greatly impacted by changes in the expression or functionality of these transporters. For instance, lower uric acid excretion and a higher risk of hyperuricemia have been linked to decreased ABCG2 activity. Similarly,, increased activity of GLUT9 and URAT1 may enhance renal urate reabsorption, thereby contributing to uric acid accumulation [24]. Along with the kidneys, the gut contributes significantly to the removal of uric acid, contributing to approximately one-third of the total uric acid excretion. The concept of the intestine as a “third kidney” has resulted from this fact. Urate transporters such as ABCG2 are expressed by intestinal epithelial cells and help secrete uric acid into the gut lumen, where gut microbes can further metabolize it [15,25]. In situations of renal impairment, where intestinal excretion may compensate for decreased renal clearance, this alternate excretory pathway becomes more crucial [25]. The principal pathways of uric acid synthesis and metabolism, together with intestinal and renal urate elimination and the major sites of action of xanthine oxidase inhibitors, are summarized in Figure 2.

### 2.2. Hyperuricemia and Dysbiosis: Cause or Consequence?

The relationship between gut microbiota and hyperuricemia is complex and bidirectional, raising the question of whether dysbiosis is a cause or a consequence of elevated uric acid levels. Increasing evidence supports the notion that gut microbiota may participate in uric acid metabolism and systemic metabolic regulation [29]. Patients with hyperuricemia have been reported to exhibit alterations in gut microbial composition, characterized by reduced α-diversity and shifts in β-diversity compared to healthy individuals [29,30]. These changes often include a decrease in beneficial bacterial genera, such as *Lactobacillus* and *Bifidobacterium*, alongside an increase in opportunistic or pro-inflammatory taxa, including members of the *Enterobacteriaceae* family. Such compositional shifts are associated with altered metabolic capacity of the microbiome [30]. Gut microbiota may influence uric acid metabolism through multiple mechanisms. Certain microbial species possess enzymes capable of degrading purines or uric acid, thereby potentially contributing to intestinal purine or urate metabolism. Conversely, dysbiosis may lead to increased production of uric acid precursors or reduced degradation capacity [31]. However, these effects are not uniform across microbial taxa and may vary according to strain, substrate availability, and the surrounding microbial ecosystem [31,32].

Microbial metabolites play a central role in mediating host–microbe interactions. Short-chain fatty acids, including acetate, propionate, and butyrate, are produced through the fermentation of dietary fibers and exert beneficial effects on host metabolism. These metabolites support intestinal barrier function, regulate immune responses, and may indirectly influence uric acid handling [32]. However, evidence for a direct urate-lowering effect of SCFAs in humans, remains limited. In contrast, metabolites such as trimethylamine N-oxide (TMAO) have been associated with adverse cardiovascular outcomes and may exacerbate metabolic dysfunction [33]. Indole derivatives produced from tryptophan metabolism also contribute to immune regulation and epithelial integrity [34]. However, the directionality of the relationship between dysbiosis and hyperuricemia remains unclear. Elevated uric acid levels may alter the gut environment, favoring the growth of certain microbial populations, whereas preexisting dysbiosis may predispose individuals to impaired uric acid metabolism (Table 1) [32].

### 2.3. Intestinal Barrier Dysfunction and Endotoxemia

**Figure 3:**
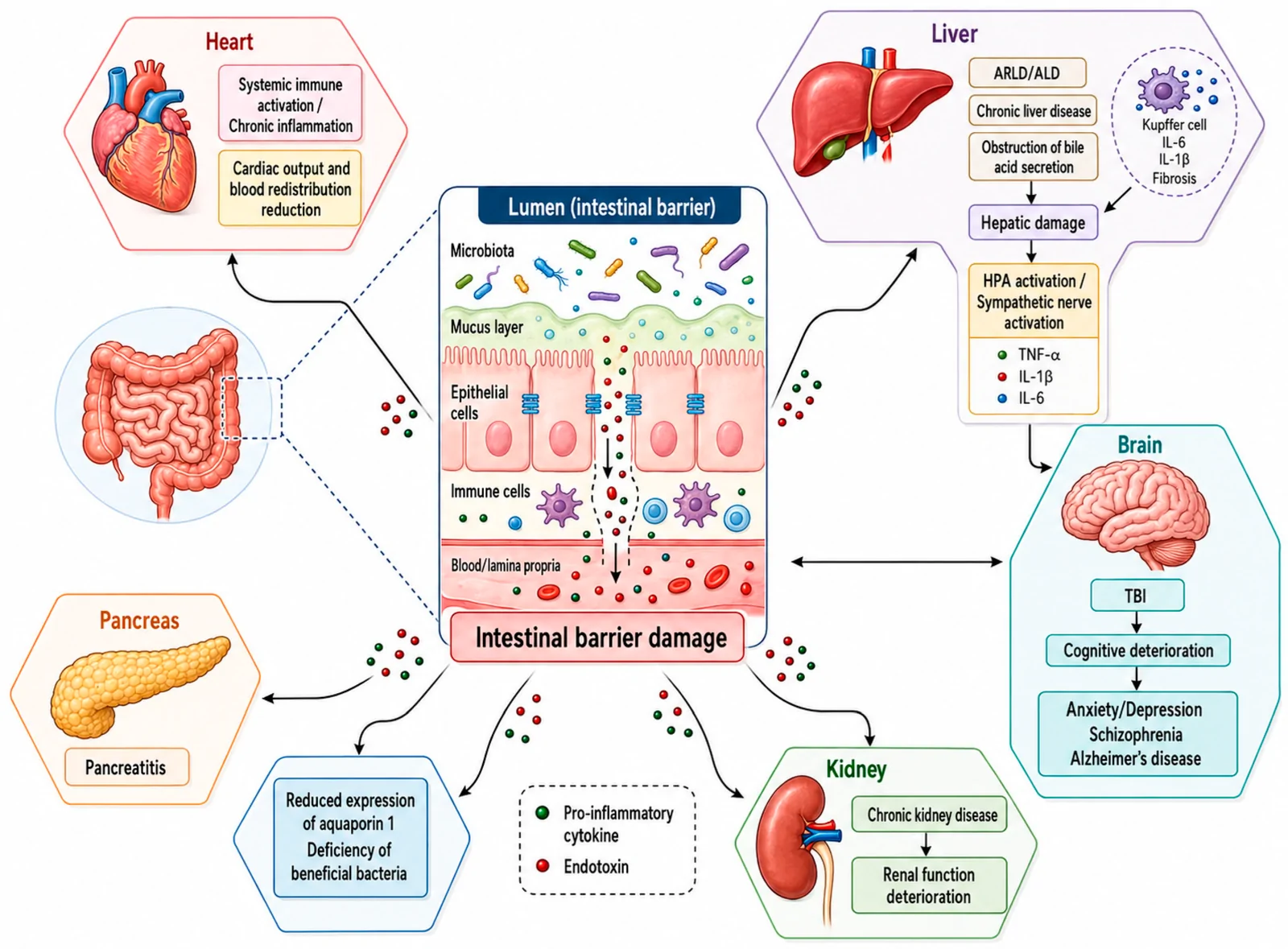
Intestinal barrier damage in the “gut–organ” axes [35]. Green circles represent pro-inflammatory cytokines, red circles represent endotoxin, and arrows indicate the proposed interactions between the intestine and the respective organs. The different colors surrounding the organ panels are used for visual distinction only and do not represent specific experimental groups or quantitative variables.

The integrity of the intestinal barrier is essential for maintaining host homeostasis and preventing systemic exposure to microbial components. In hyperuricemia, preclinical and mechanistic evidence suggests that intestinal barrier function may be compromised and associated with systemic inflammation and metabolic disturbances (Figure 3) [15,32]. The intestinal barrier is primarily maintained by tight junction proteins, including occludin, claudin-1, and zonula occludens-1 (ZO-1), which regulate paracellular permeability. In experimental hyperuricemia models, reduced expression of these proteins has been reported, leading to increased intestinal permeability, commonly referred to as “leaky gut”. This disruption allows the translocation of bacterial endotoxins, particularly lipopolysaccharides, into the bloodstream. Elevated circulating levels of lipopolysaccharides can trigger the activation of pattern recognition receptors, such as Toll-like receptor 4 (TLR4), initiating downstream signaling cascades [35]. One of the key pathways activated by LPSs is the nuclear factor kappa B (NF-κB) signaling pathway, which regulates the expression of pro-inflammatory cytokines including tumor necrosis factor-alpha (TNF-α), interleukin-1 beta (IL-1β), and interleukin-6 (IL-6) [35]. This systemic inflammatory response may contribute to metabolic dysfunction and exacerbate renal and cardiovascular complications associated with hyperuricemia. Furthermore, inflammation-induced oxidative stress can impair renal function and alter urate transporter expression, further disrupting uric acid homeostasis [15,32]. Thus, intestinal barrier dysfunction may represent an important mechanistic link between gut microbiota alterations and systemic metabolic disturbances [32,35].

## 3. Multitarget Mechanisms of Probiotics in Hyperuricemia Alleviation

Probiotic interventions have been investigated for their potential uric acid-lowering effects through a complex network of interconnected mechanisms that extend beyond a single metabolic pathway. Although established urate-lowering drugs primarily target uric acid production or excretion, probiotic-based interventions have been proposed to influence multiple processes, including purine metabolism, intestinal excretion, gut microbiota composition, inflammatory signaling, and renal function. These proposed multitarget effects support their investigation as emerging adjunctive strategies for hyperuricemia; however, evidence for several individual mechanisms remains predominantly preclinical [36].

### 3.1. Modulation of Purine Metabolism

One proposed mechanism through which probiotics may influence uric acid levels is the modulation of purine metabolism, particularly at the level of enzymatic degradation and intestinal absorption.

#### 3.1.1. Xanthine Oxidase (XO) Inhibition

Xanthine oxidase is a key enzyme in purine metabolism, responsible for the conversion of hypoxanthine to xanthine and subsequently to uric acid. Inhibition of XO is the principal mechanism of action of commonly used drugs, such as allopurinol. Interestingly, preclinical studies have reported XO-modulating effects of certain probiotic strains or probiotic-derived products [37]. For example, probiotic-derived products from *Lacticaseibacillus rhamnosus* have been associated with reduced XO activity in experimental hyperuricemia models [37]. This inhibition may occur through the production of bioactive metabolites, including peptides and organic acids, which may influence XO activity or expression. Additionally, certain microbial metabolites may act as competitive substrates, thereby reducing uric acid formation. These mechanisms should currently be regarded as preclinical or mechanistic evidence rather than established effects in human hyperuricemia. However, the magnitude of XO inhibition by probiotics is generally lower than that achieved by pharmacological inhibitors, raising questions about clinical relevance. Furthermore, most studies rely on simplified enzyme assays, which may not accurately reflect in vivo conditions [37].

#### 3.1.2. Adenosine Deaminase (ADA) Regulation

Adenosine deaminase catalyzes the deamination of adenosine to inosine, a precursor in purine degradation. Dysregulation of ADA activity can influence purine flux and uric acid production [38]. The potential influence of probiotics on ADA activity through changes in microbial metabolism has been proposed; however, direct evidence demonstrating probiotic-mediated regulation of ADA in hyperuricemia remains limited. Therefore, this pathway should currently be considered a mechanistic hypothesis rather than an established probiotic effect. Although less studied than XO inhibition, this mechanism represents a potentially relevant regulatory node in purine metabolism [38].

#### 3.1.3. Nucleoside Transport and Purine Availability

Another proposed mechanism involves the reduction of the intestinal availability or absorption of purines and nucleosides. However, direct evidence that probiotics inhibit host intestinal nucleoside transporters in hyperuricemia remains limited, and this mechanism should therefore be interpreted cautiously. Additionally, certain probiotic strains can metabolize purines directly within the gut lumen, potentially reducing the amount of purine substrate available for absorption and subsequent uric acid production [39].

### 3.2. Enhancement of Intestinal Uric Acid Excretion

In addition to their potential effects on uric acid production, probiotics may influence urate elimination through intestinal pathways; however, supporting evidence is predominantly preclinical.

#### 3.2.1. ABC Transporter Regulation

ATP-binding cassette transporters, particularly ABCG2 and ABCC2, play a central role in uric acid secretion into the intestinal lumen [24]. Direct preclinical evidence indicates that specific probiotic strains can enhance intestinal ABCG2 expression. For example, *Lactiplantibacillus plantarum* 06CC2 increased intestinal ABCG2 expression and fecal uric acid excretion in a mouse model [40]. Evidence for comparable probiotic-mediated regulation of ABCC2 is less established. This effect may be mediated through microbial metabolites, such as short-chain fatty acids, which may influence gene expression via epigenetic and signaling pathways. Enhanced intestinal ABCG2 activity may increase intestinal urate excretion; however, whether probiotic-induced transporter regulation provides clinically meaningful benefits in patients with impaired kidney function remains to be established [40].

#### 3.2.2. Tight Junction Restoration

Probiotics contribute to the restoration of intestinal barrier integrity by upregulating tight junction proteins such as occludin, claudin-1, and ZO-1 in several experimental and gastrointestinal disease models [41]. Improved barrier function reduces paracellular permeability and limits the translocation of luminal microbial products. Moreover, enhanced barrier integrity limits endotoxin translocation, thereby reducing inflammation and potentially influencing metabolic processes relevant to uric acid homeostasis [41]. However, most evidence supporting probiotic-mediated tight junction restoration derives from intestinal, inflammatory, or metabolic models rather than direct human studies of hyperuricemia.

#### 3.2.3. Colonic Uric Acid Secretion

The colon is increasingly recognized as an important site of uric acid excretion. Probiotics have been proposed to influence this pathway by modulating epithelial transport mechanisms and changing the luminal environment to promote uric acid secretion. Furthermore, gut microbiota may transform uric acid into allantoin or other metabolites through microbial urate-metabolizing pathways [15]. This microbial contribution highlights the role of the intestine as an active excretory organ [15]. However, the extent to which probiotic supplementation enhances colonic urate secretion in humans remains insufficiently established.

### 3.3. Gut Microbiota Restructuring

A central mechanism of probiotic action is the modulation of gut microbiota composition and function. Probiotic supplementation can influence the abundance and activity of beneficial bacterial genera, such as *Lactobacillus*, *Bifidobacterium*, and *Akkermansia*. These bacteria contribute to improved metabolic homeostasis through production of SCFAs, maintenance of epithelial integrity, and regulation of immune responses [42]. At the same time, they may suppress potentially pathogenic or pro-inflammatory taxa, including some members of the *Enterobacteriaceae* family. This suppression may occur through competitive exclusion, production of antimicrobial compounds, and modulation of the intestinal environment [43]. Importantly, microbial effects on purine and urate metabolism are strain-, substrate-, and ecosystem-dependent; therefore, individual taxa should not be classified uniformly as “uric acid-producing” or “uric acid-lowering”.

#### Functional Gene Modulation

Functional gene modulation has been proposed as a mechanism through which probiotics may influence metabolic processes relevant to hyperuricemia. Unlike simple compositional shifts in gut microbiota, this mechanism may involve dynamic alterations in the genetic and transcriptional activity of both microbial communities and host cells, ultimately reshaping metabolic outputs relevant to uric acid homeostasis [44]. At the microbial level, genes involved in purine metabolism, including those responsible for de novo synthesis and catabolic pathways, represent potentially relevant functional targets. Key genes such as xdh (encoding xanthine dehydrogenase), guaA (GMP synthase), and purH/purF (enzymes in de novo purine biosynthesis) are particularly relevant. Downregulation of xdh reduces the enzymatic conversion of hypoxanthine to xanthine and subsequently to uric acid, thereby directly limiting uric acid production. Similarly, modulation of guaA and other purine biosynthetic genes can decrease the intracellular pool of purine nucleotides, further restricting substrate availability for uric acid generation [45]. However, direct evidence that probiotic interventions consistently regulate these specific genes in hyperuricemia remains limited; therefore, these gene-level relationships should primarily be interpreted a mechanistic context rather than established probiotic effects.

In parallel, probiotic-induced modulation of the gut microbiome has been proposed to influence microbial metabolic activity and host energy metabolism., which recycles purine bases such as hypoxanthine and guanine. This shift could theoretically reduce the formation of uric acid as a byproduct. Direct evidence demonstrating that probiotics consistently induce this metabolic reprogramming in hyperuricemia remains limited [46]. Beyond microbial genes, probiotics may also influence host gene expression, particularly genes encoding urate transporters and regulators of epithelial function. Short-chain fatty acids (SCFAs), especially butyrate, may act as mediators in this process. Through mechanisms such as histone deacetylase inhibition and epigenetic remodeling, SCFAs have been proposed to influence the expression of transporters such as ABCG2, which facilitates uric acid excretion into the intestinal lumen. Concurrently, probiotics may downregulate reabsorptive transporters such as URAT1, thereby promoting net uric acid elimination [15]. These transporter-related effects are supported mainly by preclinical evidence and should not yet be generalized to human hyperuricemia. In addition, probiotic-induced metabolites can activate intracellular signaling pathways, including AMPK and peroxisome proliferator-activated receptors (PPARs), which further regulate genes involved in energy metabolism, inflammation, and urate handling. These pathways provide plausible mechanistic links between microbial metabolites and host metabolism, although their direct contribution to urate lowering in humans remains uncertain.

High-throughput omics technologies, including metagenomics and metatranscriptomics, areimportant tools for investigating functional changes in the microbiome [47]. These approaches can identify alterations in genes involved in purine metabolism, SCFA production, epithelial interactions, and inflammatory pathways. However, direct evidence that probiotic interventions consistently produce these changes in hyperuricemia remains limited. Therefore, functional analysis can complement taxonomic profiling by identifying metabolic changes that may not be apparent from the microbial composition alone [47]. Furthermore, microbial metabolites, such as SCFAs, indole derivatives, and polyamines, act as signaling molecules that reinforce gene-level regulation. SCFAs enhance epithelial integrity and modulate immune responses, whereas indole derivatives activate aryl hydrocarbon receptor (AhR) signaling, contributing to mucosal homeostasis. Polyamines support cellular repair and proliferation, indirectly influencing the intestinal barrier function. These effects provide relevant mechanistic context, but most have not been directly validated as urate-lowering mechanisms in human hyperuricemia [48].

### 3.4. Anti-Inflammatory and Antioxidant Effects

Since this article focuses on probiotic-based strategies in hyperuricemia rather than immunology alone, the anti-inflammatory and antioxidant mechanisms are summarized in a focused manner to avoid becoming overexpanded and repetitive.

Chronic inflammation and oxidative stress are closely associated with hyperuricemia and its related complications. Probiotic interventions have demonstrated anti-inflammatory and antioxidant effects in experimental and other metabolic or inflammatory models; however, the extent to which these effects directly contribute to urate-lowering in human hyperuricemia remains uncertain.

#### 3.4.1. NLRP3 Inflammasome Inhibition

In brief, monosodium urate crystal deposition, particularly in gout, can activate the NLRP3 inflammasome signaling and promote IL-1β-driven inflammation [49]. Endotoxemia and gut barrier impairment may also contribute to inflammatory priming; however, their direct contribution to NLRP3 activation in human hyperuricemia remains less well established [50,51]. Probiotic interventions and postbiotic preparations may indirectly influence this pathway by reducing exposure to LPSs, supporting epithelial integrity, limiting ROS generation, and attenuating signals involved in NLRP3 priming and activation [51,52]. These effects are supported mainly by preclinical and mechanistic evidence rather than direct clinical evidence in hyperuricemia. This focused interpretation highlights the biological significance of the pathway without repeating the fine details of the immunological cascades.

#### 3.4.2. Reduction in Oxidative Stress

**Figure 4:**
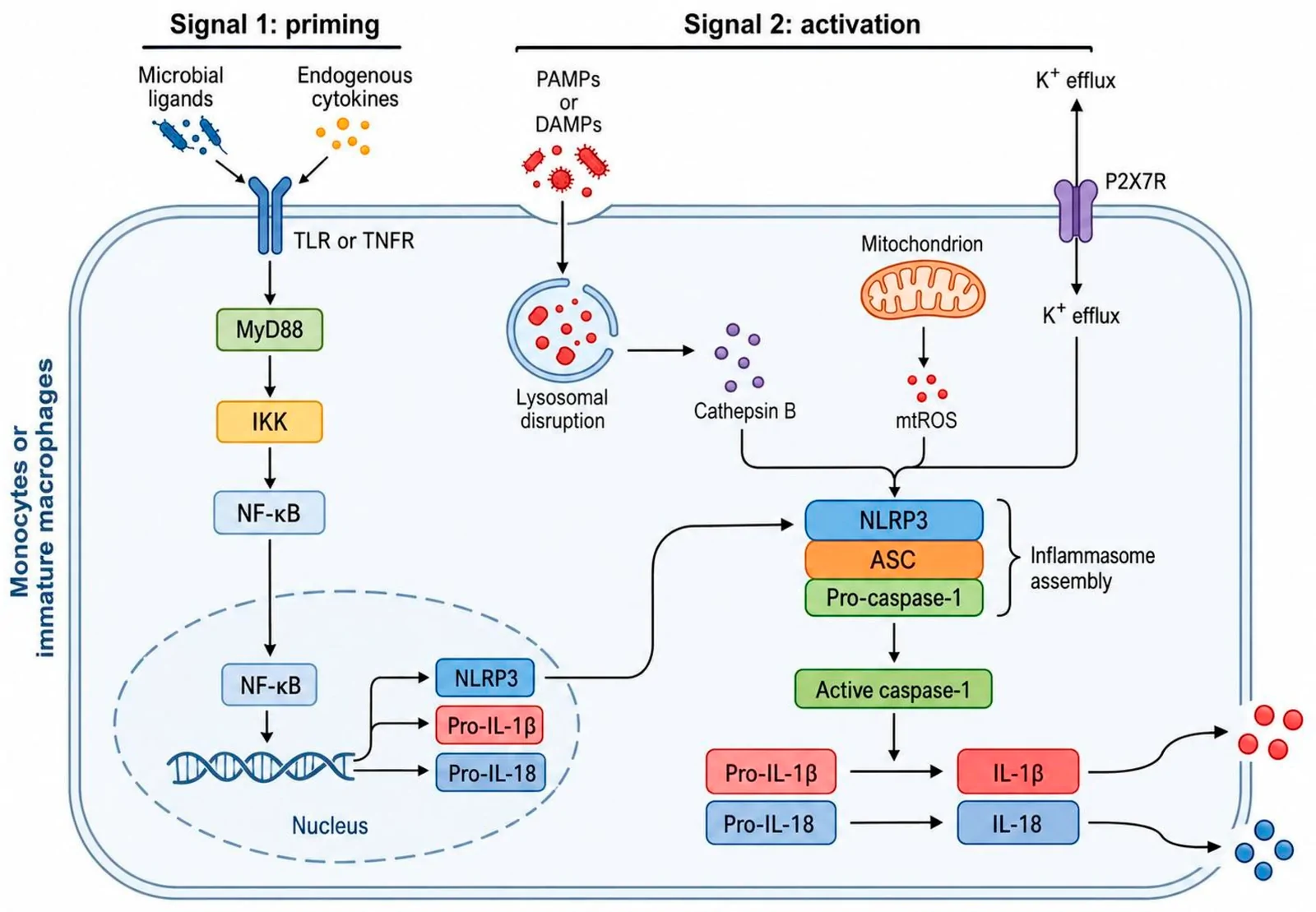
Signal pathway for NLRThe pathway involves two sequential steps: priming (Signal 1) and activation (Signal 2). During priming, microbial ligands or endogenous cytokines activate TLR/TNFR-dependent MyD88–IKK–NF-κB signaling, promoting the expression of NLRP3, pro-IL-1β, and pro-IL-18. During activation, stimuli including PAMPs/DAMPs, lysosomal disruption with cathepsin B release, mitochondrial reactive oxygen species (mtROS), and K^+^ efflux through P2X7R promote assembly of the NLRP3–ASC–pro-caspase-1 inflammasome. Caspase-1 activation subsequently mediates the maturation and release of IL-1β and IL-18. In the schematic, blue lightning symbols represent microbial ligands, orange star-like symbols represent endogenous cytokines, and red star-like symbols represent PAMPs/DAMPs. Blue, orange, green, yellow, light-blue, and red boxes are used to distinguish NLRP3, ASC, caspase-1-related components/MyD88, IKK, NF-κB/IL-18-related components, and IL-1β-related components, respectively. Black arrows indicate the direction of signaling, activation, processing, or release, and the dashed oval indicates the nucleus. Colors are used for visual distinction and do not represent quantitative differences.

Oxidative stress is closely associated with hyperuricemia and its associated complications, including renal dysfunction, endothelial injury, and chronic inflammation. It arises from an imbalance between the excessive production of reactive oxygen species (ROS) and the capacity of antioxidant defense systems to neutralize them. In experimental hyperuricemia, oxidative stress may both accompany and contribute to disturbances associated with elevated uric acid levels, potentially creating a self-amplifying pathological cycle [50]. At the molecular level, uric acid metabolism itself contributes to oxidative stress. The enzyme xanthine oxidase, which catalyzes the conversion of hypoxanthine to xanthine and subsequently to uric acid, generates superoxide anions and hydrogen peroxide as byproducts. Increased xanthine oxidase activity, therefore, not only elevates uric acid production but also enhances ROS generation. Additionally, monosodium urate crystals can promote mitochondrial dysfunction and stimulate NADPH oxidase activity in immune cells, further amplifying oxidative stress [50]. Within the scope of this review, the antioxidant effect of probiotics may be summarized as enhancement of endogenous antioxidant defenses, reduction in ROS accumulation and indirect attenuation of redox-sensitive inflammatory signaling. These effects provide relevant mechanistic context for intestinal and renal protection and may indirectly support urate homeostasis; however, their direct contribution to urate lowering in humans remains insufficiently established (Figure 4) illustrates the two-step activation of the NLRP3 inflammasome, highlighting the contribution of mitochondrial reactive oxygen species (mtROS) to inflammasome assembly and subsequent inflammatory cytokine maturation. This pathway provides a mechanistic link between oxidative stress and inflammation, although its modulation by probiotics in hyperuricemia requires further investigation.

#### 3.4.3. Cytokine Profile Normalization

Probiotic interventions have been reported to modulate cytokine profiles in experimental and inflammatory models, including reductions in pro-inflammatory mediators, such as IL-6 and TNF-α, and increases in anti-inflammatory cytokines, such as IL-10 [51]. Such cytokine changes may contribute to reduced inflammatory activity and tissue injury; however, direct evidence that these effects mediate urate lowering in human hyperuricemia remains limited [51].

### 3.5. Renal Protection and Uric Acid Handling

Thus, to avoid repetition of the preceding discussion on the gut–kidney–liver axis and urate transporters, only a brief summary of the renal dimensions is provided here. The kidneys play a central role in uric acid excretion, and renal dysfunction is both a cause and a consequence of hyperuricemia [23,24]. Probiotics may exert nephroprotective effects that could indirectly support uric acid handling; however, much of the evidence for probiotic-mediated renal protection derives from preclinical studies or renal conditions other than hyperuricemia [52].

#### 3.5.1. Nephroprotective Effects

The potential nephroprotective effects of probiotics relevant to hyperuricemia can be summarized as three interrelated processes: suppression of tubular damage, alleviation of oxidative and inflammatory injuries, and partial recovery of gut–kidney signaling. Probiotics may help attenuate this cycle by maintaining renal tissue integrity and reducing the inflammatory load, which, in turn, may support renal function and urate handling [52]. Direct evidence that these nephroprotective effects lower serum urate in human hyperuricemia remains limited. Hence, renal protection is best understood as a downstream functional consequence of the other mechanisms discussed above (such as microbiota rebalancing, barrier support, SCFA generation, and lower systemic inflammation), and is not a completely separate mechanism that needs to be repeatedly explained from the same pathways.

Probiotic interventions may contribute to renal protection by reducing oxidative stress, a major component of kidney damage. Increased reactive oxygen species production in renal tissues is linked to hyperuricemia, which can cause lipid peroxidation, mitochondrial malfunction, and cellular death [50]. By boosting antioxidant defenses and increasing the activity of enzymes such as glutathione peroxidase and superoxide dismutase, probiotic supplementation has been reported to reduce oxidative stress and support renal cellular integrity in experimental and other renal disease settings [52]. Another important mediator of renal damage caused by hyperuricemia is inflammation. Pro-inflammatory cytokines, including IL-1β, IL-6, and TNF-α, are released when inflammatory pathways, such as NF-Κb and NLRP3 inflammasome signaling, are activated. These cytokines can contribute to tubular injury, renal fibrosis, and a gradual decline in kidney function [51,53]. Probiotic interventions have shown anti-inflammatory effects in experimental and renal disease models, including reductions in inflammatory mediators; however, direct evidence that probiotics inhibit NF-κB or NLRP3 signaling specifically in human hyperuricemia remains limited [51,52]. Reduced endotoxemia and changes in gut microbiota may contribute to these indirect anti-inflammatory effects. Additionally, new research indicates that metabolites generated from probiotics, such as short-chain fatty acids, may protect kidney tissues. These metabolites may improve mitochondrial performance, lower inflammation, and alter the expression of genes linked to cellular repair and survival. SCFA-associated signaling has been linked to pathways including AMPK and Nrf2 in experimental settings; however, their direct role in probiotic-mediated nephroprotection in hyperuricemia has not been established. From a physiological perspective, preservation of renal function could support urate elimination. The kidneys can effectively remove urate if the glomerular filtration rate and tubular transport capacity are maintained. This provides a plausible indirect link between renal protection and urate homeostasis; however, it should not be interpreted as direct evidence that probiotic nephroprotection lowers serum urate in humans [23,24,52].

#### 3.5.2. Regulation of Renal Transporters

In this section, details of transporters are not repeated extensively to prevent redundancy. To summarize, URAT1 and GLUT9 are major urate transporter transporters, whereas ABCG2 contributes to urate excretion [24]. Preclinical studies suggest that microbiota-directed interventions may influence urate-transporter expression; however, direct evidence for consistent probiotic-mediated regulation of renal URAT1, GLUT9, and ABCG2 in human hyperuricemia remains limited. The following is a brief overview that should be read alongside the earlier discussion on urate transport in the gut–kidney–liver axis.

Probiotics may indirectly affect renal transporter performance through their anti-inflammatory and antioxidant properties. Renal function is known to be hampered by oxidative stress and chronic inflammation, which may also alter transporter expression. Inflammatory signaling may influence renal urate handling and transporter expression, although the specific contribution of individual cytokines to probiotic-mediated transporter regulation requires further validation. Probiotic interventions may indirectly support renal function by reducing inflammation and oxidative stress, but the evidence linking these effects specifically to the normalization of urate transporter expression in hyperuricemia remains predominantly mechanistic or preclinical [54].

## 4. Synergistic Strategies: Beyond Single-Strain Probiotics

Although single-strain probiotics have shown potential in preclinical and limited clinical studies of hyperuricemia, strain-specific activity, environmental sensitivity, and insufficient metabolic coverage maylimit their effectiveness [36,44]. Because uric acid dysregulation is complex, there is increasing awareness that synergistic approaches that incorporate several functional elements may provide broader functional coverage than single-component approaches. These strategies use complementary pathways to improve host interactions, metabolic output, and microbial survival, with the potential to influence several processes relevant to uric acid homeostasis [54]. However, evidence that these synergistic strategies are clinically superior to single-strain probiotics or established urate-lowering therapies remains limited.

### 4.1. Synbiotics: Probiotics + Prebiotics

Synbiotics comprise live microorganisms together with substrate(s) selectively utilized by host microorganisms that confer a health benefit [12,54]. Prebiotics are substrates selectively utilized by host microorganisms that confer a health benefit [11,54]. Commonly used prebiotics include fructooligosaccharides (FOS) and galactooligosaccharides (GOS)), inulin, and resistant starch. These compounds resist digestion in the upper gastrointestinal tract and fermented by gut microbiota in the colon, leading to the production of short-chain fatty acids and other bioactive metabolites [54]. The synergy between probiotics and prebiotics may occur through several mechanisms.

First, prebiotics serve as selective substrates that promote the growth and metabolic activity of administered probiotic strains. This may enhance their persistence and functional activity in the gut. Second, fermentation of prebiotics leads to increased production of short-chain fatty acids (SCFAs), particularly butyrate, which plays a central role in regulating intestinal barrier integrity and inflammation [54]. SCFAs may also influence gene expression through epigenetic mechanisms; however, their direct contribution to urate transport in human hyperuricemia remains insufficiently established. Third, synbiotics create a favorable intestinal microenvironment by lowering pH and suppressing pathogenic bacteria, thereby potentially reducing endotoxin exposure and influencing metabolic processes relevant to urate homeostasis [54]. Direct evidence for the urate-lowering efficacy of synbiotics in human hyperuricemia remains limited.

### 4.2. Postbiotics: Inanimate Microbial Preparations and Their Components

Postbiotics are a new and growing research field within functional foods and are defined as preparations of inanimate microorganisms and/or their components that confer health benefits on the host [13]. Such preparations may contain inactivated microbial cells and cellular components, including cell-wall structures, and may also contain microbial metabolites when these remain associated with the preparation [13,55,56]. Isolated metabolites alone should not be automatically classified as postbiotics. Unlike live probiotics, the biological effects of postbiotics are not dependent on their viability. Postbiotic preparations may offer practical advantages, including improved storage stability and greater opportunities for standardization [46,56]. They have also been proposed as potentially safer alternatives to administering viable microorganisms in some settings; however, safety remains preparation- and host-dependent and should not be assumed universally, particularly in vulnerable populations [13,55]. These characteristics may address some practical limitations associated with live probiotic formulations.

### 4.3. Coadministration with Bioactive Compounds

Combining probiotics with bioactive dietary The combination of represents another potential synergistic strategy. These combinations exploit complementary biochemical properties to potentially enhance their functional effects; however, evidence for superior therapeutic efficacy in hyperuricemia remains limited.

#### 4.3.1. Polyphenol–Probiotic Synergy

The combination of polyphenols and probiotics represents a potential synergistic approach relevant to hyperuricemia research, driven by complementary interactions at both the microbial and host levels. Polyphenols, a diverse class of plant-derived secondary metabolites, including flavonoids, phenolic acids, anthocyanins, and curcuminoids, are well known for their antioxidant, anti-inflammatory, and enzyme-modulating properties. However, their biological availability can be limited by poor bioavailability, rapid metabolism, and low intestinal absorption. Microbial biotransformation may modify the bioavailability and biological activity of polyphenols, providing a mechanistic basis for studying probiotic–polyphenol interactions [57,58]. At the gut level, probiotics may contribute to the biotransformation of polyphenols into bioactive metabolites. Many dietary polyphenols are present in complex glycosylated or polymeric forms that are poorly absorbed in the small intestine. Probiotic bacteria, particularly species within *Lactobacillus* and *Bifidobacterium*, possess enzymatic systems such as β-glucosidases, esterases, and decarboxylases that can convert these compounds into smaller, more bioavailable phenolic metabolites [56,57]. These metabolites may differ from their parent compounds in terms of bioavailability and biological activity. However, evidence that probiotic-mediated polyphenol biotransformation directly enhances urate lowering remains limited. Certain polyphenol-derived compounds have demonstrated urate-lowering and XO-related effects in experimental animal models [58]. A recent systematic review and meta-analysis reported reductions in serum uric acid across animal studies of selected polyphenols, although substantial heterogeneity and methodological limitations restrict direct clinical translation [58]. Conversely, polyphenols exert prebiotic-like effects that may promote the growth of beneficial gut bacteria. They have been associated with increases in the abundance of probiotic genera such as *Lactobacillus*, *Bifidobacterium*, and *Akkermansia* while potentially suppressing pathogenic or pro-inflammatory taxa, including members of the *Enterobacteriaceae* family. This bidirectional interaction provides a plausible basis for reciprocal interactions in which gut microorganisms metabolize polyphenols while polyphenols influence microbial ecology [57]. At the molecular level, polyphenol–probiotic synergy has been proposed to involve the modulation of key signaling pathways. Polyphenols and their metabolites can activate antioxidant pathways such as Nrf2, leading to upregulation of endogenous antioxidant enzymes and reduction in oxidative stress. Simultaneously, they may inhibit inflammatory pathways including NFκB and the NLRP3 inflammasome, thereby reducing the production of pro-inflammatory cytokines such as IL1β and TNFα. Whether these molecular effects are consistently amplified by coadministration with probiotics in hyperuricemia has not been established and should currently be regarded as a mechanistic hypothesis [36]. Furthermore, polyphenols may influence urate transport mechanisms. Emerging evidence suggests that certain polyphenols can modulate reabsorptive transporters such as URAT1 and upregulate excretory transporters like ABCG2, thereby potentially influencing urate handling in experimental models [58]. Direct evidence that probiotics potentiate polyphenol-induced regulation of these urate transporters remains limited. Another critical aspect of this synergy involves the improvement in intestinal barrier integrity. Polyphenols strengthen tight junctions and reduce epithelial permeability, while probiotics enhance mucosal defense and SCFA production. These complementary effects may reduce lipopolysaccharide translocation and inflammatory signaling; however, their combined contribution to uric acid homeostasis remains predominantly mechanistic and preclinical [58,59].

#### 4.3.2. Peptide–Probiotic Combinations

The integration of bioactive peptides with probiotics represents an emerging synergistic strategy with potential relevance to the treatment of hyperuricemia. Bioactive peptides, typically derived from the enzymatic hydrolysis of dietary proteins or microbial fermentation, possess a wide range of biological activities, including antioxidant, anti-inflammatory, and enzyme-inhibitory effects. Combining bioactive peptides with probiotics may provide complementary functional effects; however, direct evidence demonstrating the enhanced efficacy of peptide–probiotic combinations in hyperuricemia remains limited [19,59,60].

One of the most important contributions of bioactive peptides at the biochemical level is their capacity to inhibit enzymes involved in uric acid production, especially xanthine oxidase (XO). Certain peptides can interact with the XO active site and function as competitive or non-competitive inhibitors, particularly those with hydrophobic and aromatic amino acids. Consequently, less hypoxanthine and xanthine may be converted to uric acid [19,60]. Probiotics may independently influence purine-substrate availability or XO-related pathways; however, evidence that coadministration with peptides potentiates XO inhibition remains primarily mechanistic. Bioactive peptides may also indirectly influence purine breakdown pathways by influencing adenosine metabolism in addition to XO inhibition. Although modulation of purine-related enzymes has been proposed for selected food-derived peptides, direct evidence for ADA regulation by peptide–probiotic combinations in hyperuricemia remains insufficient [19,60]. The production and modification of bioactive peptides by probiotics is an essential aspect of peptide–probiotic synergy. Probiotic bacteria, such as *Lactobacillus* spp., contain proteolytic systems that are capable of breaking down food proteins into smaller peptides during fermentation, some of which may possess biological activity [59]. Compared to their parent proteins, these peptides may exhibit antioxidant and enzyme inhibitory qualities. In fermented-food systems, probiotic microorganisms can therefore function as biotransformation agents that generate or modify bioactive peptides [59]. Peptides can be broken down by digestive enzymes in the gastrointestinal tract. The extent to which probiotic coadministration protects peptides from gastrointestinal degradation or enhances their systemic bioavailability depends on the strain, peptide, and formulation and requires further direct investigation [59]. From a metabolic standpoint, peptide–probiotic combinations may have complementary effects on oxidative stress and inflammation, two processes associated with hyperuricemia. Because they scavenge reactive oxygen species and lower lipid peroxidation, bioactive peptides have reported antioxidant qualities [60]. A stronger redox equilibrium may be supported by probiotic supplementation, which can influence the activity of natural antioxidant enzymes. Probiotics may reduce upstream triggers, including endotoxemia, while peptides may modulate pro-inflammatory signaling pathways, such as NF-κB [60]. However, direct evidence that peptide–probiotic combinations suppress NLRP3 or NF-κB signaling specifically in hyperuricemia remains limited. Modulation of the gut microbiota is another significant aspect of this interaction. By functioning as antimicrobial agents or bioactive compounds, bioactive peptides can affect microbial composition. Some peptides may contribute to microbial modulation through peptides that have bacteriostatic or bactericidal activity against harmful bacteria. Probiotics, in turn, can influence microbial composition and functional activity, providing a plausible basis for complementary interactions with bioactive peptides. Combinations of peptides and probiotics may also enhance the integrity of the intestinal barrier. Probiotics can support mucosal defense and SCFA production, whereas certain peptides have been demonstrated to influence tight junction proteins such as occludin and ZO-1. Together, these effects provide a plausible mechanistic basis for reduced intestinal permeability and endotoxin translocation; however, their combined contribution to uric acid metabolism has not been directly established [61].

Emerging evidence also suggests that bioactive peptides can influence renal function. Some peptides exhibit nephroprotective effects by reducing oxidative stress and inflammation in renal tissues [62]. However, evidence that peptide–probiotic combinations directly regulate renal urate transporters or improve urate excretion remains limited. From a systems-level perspective, peptide–probiotic combinations could potentially act across multiple biological layers, including enzymatic inhibition, microbial metabolism, immune regulation, and organ-level function. This provides a rationale for further investigation of hyperuricemia; however, the concept currently remains supported mainly by mechanistic and preclinical evidence rather than comparative clinical studies [19,59,62].

#### 4.3.3. Herbal Extract Integration

Traditional herbal medicines contain bioactive compounds, such as flavonoids and alkaloids, which may influence uric acid metabolism. Microbial fermentation of herbal materials may modify phytochemical composition, improve the release or biotransformation of bioactive constituents, and influence gut microbial metabolism. However, evidence for these combined effects in hyperuricemia is currently derived mainly from experimental models rather than human clinical studies [63,64]. For example, *Lactobacillus acidophilus*-fermented dandelion has been evaluated in a mouse model of hyperuricemia. The fermented preparation reduced serum uric acid and xanthine oxidase activity and was associated with changes in renal and intestinal urate-transporter expression, including GLUT9, URAT1, OAT1, and ABCG2, together with modulation of gut microbiota and inflammatory markers [63]. Similarly, *Bacillus subtilis*-fermented *Astragalus membranaceus* reduced serum uric acid in hyperuricemic mice, attenuated renal inflammation, altered urate-transporter expression, and modified the abundance of several gut microbial taxa. These findings provide direct preclinical evidence that microbial fermentation of an herbal substrate can generate functional effects relevant to urate homeostasis [64,65]. More recently, probiotic fermentation of *Flos sophorae* was reported to increase total flavonoid content and antioxidant capacity and to enhance XO inhibition in experimental assays. In a hyperuricemia-related HK-2 cell model, selected fermented preparations reduced uric acid production, downregulated ADA and GLUT9 expression, and upregulated ABCG2 expression [64]. This evidence remains in vitro and should therefore not be interpreted as proof of therapeutic efficacy in humans.

### 4.4. Multistrain Probiotic Cocktails

Multistrain probiotic formulations aim to overcome the limitations of single-strain approaches by combining multiple strains with complementary functions [61].

#### 4.4.1. Strain Compatibility and Complementarity

The concepts of strain compatibility and functional complementarity are crucial to the effectiveness of multistrain probiotic formulations and may be relevant to their potential application in hyperuricemia. Multistrain systems, in contrast to single-strain methods, seek to combine various microbial functions into a coordinated network that may broaden functional activity. However, the type of interactions between the strains at the ecological, metabolic, and molecular levels is just as important to the success of such systems as the number of strains present [66]. The capacity of several probiotic strains to coexist without antagonistic interactions is known as strain compatibility. Microorganisms compete for resources, adhesion sites, and biological niches in the gastrointestinal environment. By producing bacteriocins, organic acids, or other antimicrobial compounds, incompatible strains may inhibit one another, reducing their viability and functional performance.To prevent competitive exclusion and support stable coexistence, thorough strain selection is crucial [66]. From a mechanistic standpoint, choosing strains with different ecological niches or metabolic preferences can improve compatibility. For instance, there may be less direct competition if one strain predominantly colonizes the small intestine while another flourishes in the colon. Similarly, strains can coexist while optimizing resource consumption due to variations in substrate utilization, such as the fermentation of carbohydrates versus the metabolism of amino acids [66]. Such functional complementarity could allow multistrain formulations to influence several processes relevant to uric acid homeostasis, including microbial metabolism, inflammation, and oxidative stress; however, direct evidence demonstrating simultaneous regulation of these pathways by multistrain probiotics in human hyperuricemia remains limited. A key mechanism underlying complementarity is metabolic cross-feeding, where metabolites produced by one strain serve as substrates for another. For instance, lactate produced by certain Lactobacillus species can be utilized by other bacteria to generate butyrate, a short-chain fatty acid with well-described anti-inflammatory and barrier-enhancing properties [40]. This sequential metabolism may increase metabolic efficiency and support the production of beneficial compounds. However, the direct contribution of such cross-feeding to urate lowering remains largely mechanistic. Additionally, complementarity extends to functional gene expression within the microbial community. Different strains may contribute distinct gene sets involved in purine metabolism, SCFA synthesis, or antioxidant pathways. The combined functional potential of a multistrain community may therefore be broader than that of an individual strain, although this depends on the strains and experimental context [66]. This concept aligns with the idea of “functional redundancy and resilience,” where overlapping functions may support stability under environmental stress [66]. Another important dimension is the interaction between probiotic strains and the host environment. Complementary strains may differentially influence host signaling pathways, such as AMPK, Nrf2, and NF-κB, which are involved in metabolic, oxidative, and inflammatory regulation. For example, one strain may primarily enhance antioxidant defenses, while another modulates immune responses, providing a plausible basis for complementary host effects. However, coordinated regulation of these pathways by multistrain formulations has not been established as a direct anti-hyperuricemic mechanism and should currently be regarded as mechanistic or indirect evidence. However, designing effective multistrain formulations presents significant challenges. Interactions between strains are highly context-dependent and can be influenced by factors such as dosage ratios, delivery systems, and host-specific microbiota composition [66]. Moreover, synergistic effects observed in vitro may not always translate to in vivo conditions due to the complexity of the gastrointestinal ecosystem [40].

#### 4.4.2. Broad-Spectrum Functional Potential

One proposed advantage of multistrain probiotic formulations is their potential to provide broader functional coverage by combining strains with different physiological and metabolic activities. Given the multifactorial nature of uric acid dysregulation, which encompasses purine metabolism, renal excretion, gut microbiota imbalance, inflammation, and oxidative stress, single-strain interventions may exert strain- and pathway-specific effects, whereas multi-strain systems may combine complementary functions. However, evidence that multistrain formulations provide superior therapeutic efficacy in human hyperuricemia is limited [66]. At the metabolic level, broader functional activity may arise through the integration of diverse capabilities across different probiotic strains. For instance, one strain may exhibit strong xanthine oxidase inhibitory activity, reducing uric acid production, whereas another enhances short-chain fatty acid production, contributing to improved intestinal barrier function and anti-inflammatory effects [40,66]. Additional strains possess purine-metabolizing activity or potentially influence pathways related to urate metabolism. These complementary activities provide a mechanistic rationale for multistrain formulations; however, a cumulative effect greater than that of the individual strains has not been consistently demonstrated in hyperuricemia [66]. A key contributor to this functional breadth is the functional redundancy and resilience within the microbial consortium. Multiple strains may share overlapping functions, such as short-chain fatty acid (SCFA) production or antioxidant activity, which may help maintain functional activity under fluctuating gastrointestinal conditions. This redundancy may improve functional resilience to environmental stress or host variability [66]. Moreover, the combined activities of multistrain formulations may influence processes across multiple anatomical sites, including the gut, liver, and kidneys, thereby reinforcing the gut–kidney–liver axis. In the intestine, they may modulate the microbiota composition, enhance barrier integrity, and promote uric acid excretion. In the liver, they may influence purine metabolism and the oxidative pathways. Indirect effects on inflammation, oxidative stress, and urate-handling pathways, including reductions in inflammation and oxidative stress, have been proposed in the kidneys. These effects should currently be interpreted mainly as mechanistic or preclinical evidence rather than as established systemic efficacy in human hyperuricemia [15,24,32]. Another important aspect is the potential of multi-strain formulations to modulate complex signaling networks. Different strains may differentially influence pathways, such as AMPK, Nrf2, NFκB, and NLRP3 inflammasome signaling. These pathways are relevant to metabolic regulation, redox balance, and inflammatory responses; however, the coordinated modulation of these pathways by multistrain probiotics has not been established as a direct anti-hyperuricemic mechanism and remains primarily a mechanistic hypothesis [50,51,52].

### 4.5. Microbial Co-Culture Systems: Emerging Platforms for Functional Fermentation and Postbiotic Development—Concept and Rationale

Microbial co-culture systems are highlighted in this review because they provide a mechanistic link between probiotic-based fermentation, microbial interactions, and the generation of potentially bioactive fermentation products. Co-culture systems are used to deliberately grow two or more compatible microorganisms together to promote complementary metabolic interactions between the partners. The design principles shift the fermentation from a single-organism fermentation to a cooperative biochemical network—sometimes resulting in a more complex metabolite profile than monoculture fermentation [14]. This concept may be relevant to hyperuricemia because several biological processes contribute to urate homeostasis, such as purine handling, intestinal urate excretion, gut barrier integrity, oxidative balance, and inflammation [15,18]. However, direct evidence demonstrating that microbial co-culture systems simultaneously regulate these processes or lower uric acid in humans remains limited.

#### 4.5.1. Metabolic Cross-Feeding and Enhanced Metabolite Production

*Lacticaseibacillus rhamnosus GG* (LGG) is one of the best-characterized probiotics, with a substantial history of clinical use and safety evaluation, and is frequently used as a model strain for studying probiotic–host interactions [67]. Although the direct uric acid-lowering activity of LGG has not been established as a major strain-specific effect, LGG has documented effects on epithelial barrier function, host–microbe interactions, and immune regulation [67]. These properties provide indirect mechanistic relevance to metabolic and inflammatory processes associated with hyperuricemia; however, theyshould not be interpreted as direct anti-hyperuricemic activity. Its well-characterized adhesion, stress-response, and host-interaction properties also make LGG a useful model for investigating combination strategies. However, compatibility and functional performance in a co-culture system depend on the partner microorganism, substrate, fermentation conditions, and food matrix; therefore, they require direct experimental evaluation [14,67].

#### 4.5.2. Interactions Between LAB and Yeast

Probiotic interventions investigated in hyperuricemia vary substantially in strain composition and formulation, ranging from single-strain preparations to multistrain and synbiotic products. This heterogeneity provides useful context for combination strategies but does not itself establish the efficacy of LAB–yeast co-culture systems [67]. Human evidence remains limited and heterogeneous and is discussed separately in the clinical evidence section. LAB–yeast interactions are better established in fermented-food systems, where cross-feeding, nutrient exchange, redox modification, and matrix conditioning can influence microbial growth, survival, and fermentation performance [14]. These interactions are strain- and matrix-dependent and should not automatically be interpreted as anti-hyperuricemic synergy. Recent food-fermentation studies provide direct evidence for the technological feasibility of combining LAB with *Kluyveromyces marxianus*. For example, co-cultivation of *Lacticaseibacillus rhamnosus* GG with *K. marxianus* KLU1 in dried kurut significantly improved LGG survival during refrigerated storage compared with LGG alone, supporting the potential of yeast–LAB interactions to enhance probiotic stability in a food matrix [68]. However, this study evaluated fermentation performance and probiotic viability rather than urate-lowering efficacy.

##### LGG–*Kluyveromyces marxianus* XZ1 Co-Culture as a Conceptual Application-Oriented Case

As an application example, an LGG–*K. marxianus* XZ1 consortium can be considered as a conceptual framework for investigating the translational potential of LAB–yeast co-culture design. In this setup, the well-established and characterized probiotic profile of *Lacticaseibacillus rhamnosus GG* (LGG) is complemented by *Kluyveromyces marxianus* XZ1, a formally identified yeast strain (CCTCC No. M2022014), whose cell-free fermentation products have recently demonstrated anti-hyperuricemic activity in experimental models [69]. The XZ1 study reported inhibition of XOD and ADA, regulation of intestinal and renal urate transporters, remodeling of gut microbiota and SCFA profiles, and improvement in intestinal-barrier and oxidative-stress parameters in a hyperuricemic mouse model [69]. Importantly, these findings were obtained using *K. marxianus* XZ1 fermentation products rather than an LGG–XZ1 co-culture. Therefore, direct anti-hyperuricemic efficacy of an LGG–XZ1 consortium has not yet been demonstrated, and the proposed combination should be regarded as a testable co-culture hypothesis rather than an established therapeutic system. Conceptually and practically, such a combination could allow investigation of dynamic metabolite exchange, cooperative growth, and shared stress responses between a bacterial probiotic and a metabolically active yeast. Evidence from LGG co-culture with another *K. marxianus* strain, KLU1, supports the technological feasibility of this type of LAB–yeast association, although strain-specific compatibility with XZ1 requires direct experimental validation [68]. Such co-culture systems may generate complex fermentation-derived preparations containing microbial cells, cellular components, organic acids, peptides, exopolysaccharides, and other metabolites. However, these products should only be described as postbiotics when they satisfy the accepted postbiotic definition, and superior anti-hyperuricemic activity compared with individual cultures must be demonstrated experimentally.

##### Advantages over Monoculture Systems

Co-culture systems may offer several potential advantages over monoculture fermentation, both from a functionality and a translation perspective. First, they expand the breadth of metabolism by bringing together complementary biosynthetic capacities. Second, they might enhance fermentation robustness, as one organism may modify environmental conditions or provide metabolites that support the other [14,68]. Third, they may generate a broader fermentation-derived metabolite profile, which can be useful if the intended effect on health is a multitarget effect, such as hyperuricemia. Whether this translates into superior urate-lowering efficacy remains to be determined experimentally. Fourth, co-culture systems are design-flexible: consortium composition, substrate selection, inoculation ratio and fermentation time can be optimized for tuning the final functional profile [14]. The principal challenges to their use are process complexity, screening for compatibility, batch-to-batch variability, and the requirement for better regulatory definition; however, this does not detract from their promise as a new functional approach.

##### Non-Conventional Yeasts and Food-Matrix Applications

The application of non-conventional yeasts further illustrates the broader potential of yeast-based functional fermentation. *Meyerozyma guilliermondii* YB1 has been evaluated as a starter for type II sourdough, where fermentation reduced fermentable carbohydrates and phytic acid and increased antioxidant activity [70]. A more recent assessment of the same strain demonstrated techno-functional properties, acid and bile tolerance, antimicrobial activity, and strain-specific biosafety characteristics, supporting its evaluation as a functional food starter [71].

Similarly, *Clavispora lusitaniae* has been experimentally used for low-alcohol fermented beverages. A recent study used *C. lusitaniae* to ferment the underexploited fruit *Carissa carandas* (karonda), producing a low-alcohol beverage that retained phenolics, ascorbic acid, and antioxidant activity during refrigerated storage [72]. These examples demonstrate the technological diversity of non-*Saccharomyces* yeasts but do not establish their probiotic or anti-hyperuricemic activity. Foodsafety evaluation is particularly important when expanding non-conventional yeast applications. Starter suitability should be established at the strain level through identification, antimicrobialresistance or antifungalsusceptibility assessment, absence of relevant virulence traits, process stability, and matrix-specific safety testing. This distinction is especially important for *C. lusitaniae*, which has food-fermentation applications but has also been reported as an opportunistic pathogen in susceptible individuals; therefore, its use in functional foods requires rigorous strain-specific biosafety assessment rather than assuming safety at the species level [73].

##### Relevance to Hyperuricemia and Translational Outlook

In combination, microbial co-culture systems offer a common ground for the key themes discussed in this review. They link probiotic science to functional fermentation and potential postbiotic development and microbial synergy to systems-level metabolic control and provide an experimental pathway to next-generation functional formulations [14]. Their relevance in the context of hyperuricemia is the potential to develop metabolite-enriched formulations designed to investigate modulation of purine metabolism, intestinal excretion, epithelial integrity, oxidative stress, and inflammatory tone. Direct support for this concept is strengthened by the recent demonstration that *K. marxianus* XZ1 fermentation products alleviate hyperuricemia through XOD/ADA inhibition and a microbiota–SCFA–gut–kidney urate-transporter axis; however, these findings should not be extrapolated to an LGG–XZ1 co-culture until the combination itself is experimentally tested [69]. Thus, co-culture systems are not an “afterthought” in this review, but an integral part of its functional food perspective and an important area for future experimental and translational research. To facilitate direct comparison among the principal synergistic strategies discussed in this review, Table 2 summarizes their main mechanisms, level of evidence, advantages, and limitations.

## 5. Representative Probiotic Strains and Experimental Models

### 5.1. Representative Probiotic Strains Relevant to Hyperuricemia

*Lactiplantibacillus plantarum* is one of the probiotic species investigated in experimental hyperuricemia. Specific strains have demonstrated urate-lowering effects through mechanisms including microbial community modulation and degradation of purine nucleosides [60]. Furthermore, certain *L. plantarum* strains can metabolize purine nucleosides, potentially reducing intestinal purine availability [74]. At the molecular level, these strains may also influence gut microbiota, promote barrier integrity, and decrease oxidative stress and inflammatory signaling. In addition, L. plantarum is able to produce bioactive metabolites; short-chain fatty acids (SCFAs) and peptides may contribute to host metabolic and intestinal functions relevant to urate homeostasis [74]. These effects are strain-specific and should not be generalized to all *L. plantarum* strains. *Lacticaseibacillus rhamnosus* GG (LGG) is one of the best-characterized probiotics, with a substantial history of clinical use and safety evaluation, and has frequently been used as a model strain for probiotic–host interactions [74]. Direct uric acid-lowering activity of LGG has not been established as a major strain-specific effect. However, LGG has documented effects on epithelial barrier function, host–microbe interactions, and immune regulation [67]. These effects provide indirect mechanistic relevance to hyperuricemia but should not be interpreted as direct anti-hyperuricemic efficacy. Most relevantly, this strain is highly adaptable and has well-characterized adhesion and host-interaction properties, supporting its investigation for combination approaches, such as synbiotics and co-culture systems [74]. *Akkermansia muciniphila* is considered a next-generation probiotic candidate, gaining attention for its role in metabolic health. This bacterium specializes in mucin degradation and has been associated with support of the intestinal barrier, reduction in metabolic inflammation, and improvement in insulin sensitivity [75]. In hyperuricemia, its role is primarily indirect, focusing on mechanisms related to intestinal and metabolic homeostasis rather than an established direct urate-lowering effect. Direct evidence for *A. muciniphila* as an intervention in human hyperuricemia remains limited.

### 5.2. In Vitro Screening Methodologies

The identification of effective probiotic strains requires comprehensive in vitro screening approaches that evaluate both enzymatic activity and cellular interactions. Such assays are useful for identifying candidate strains but should not be interpreted as evidence of anti-hyperuricemic efficacy in vivo.

#### 5.2.1. Purine Degradation Capacity Assessment

One of the primary screening methods involves evaluating the ability of probiotic strains to degrade purine compounds, such as adenine, guanine, hypoxanthine, and xanthine. This is typically assessed using chromatographic techniques to quantify the substrate depletion and metabolite formation [74]. Strains with high purine degradation capacity can reduce intestinal purine availability, thereby lowering uric acid production. A high in vitro purine degradation capacities may indicate the potential to reduce intestinal purine availability; however, the resulting effect on systemic uric acid levels requires confirmation in appropriate animal models [74].

#### 5.2.2. Xanthine Oxidase Inhibitory Activity Assays

Xanthine oxidase (XO) inhibitory activity assays are commonly used in vitro screening methods for evaluating the XO inhibitory potential of probiotic strains and their metabolites. Given that xanthine oxidase catalyzes the final steps of purine degradationby converting hypoxanthine to xanthine and subsequently to uric acid, its inhibition is a well-established therapeutic target for the management of hyperuricemia [37]. These assays were designed to quantify the ability of probiotic-derived compounds to suppress XO enzymatic activity under controlled conditions, providing an initial indication of their potential to reduce uric acid production. However, XO inhibition observed in vitro does not necessarily predict urate-lowering efficacy in vivo and should validated in cellular and animal models.

#### 5.2.3. Cellular Models: Caco-2 and HK-2 Cells

Cell-based models provide a more physiologically related platform for mechanisms relevant to probiotic and urate-related interventions than simplified enzyme assays. •Caco-2 cells (intestinal epithelial model)were used to study intestinal absorption, barrier integrity, and transporter expression, including intestinal ABCG2-mediated urate transport [76].

•HK-2 cells (renal proximal tubular cells)→ used to assess urate transport, oxidative stress, and inflammatory responses and the expression of renal urate transporters such as GLUT9, URAT1, ABCG2, and OAT3 [77].

These models allow investigation of urate transporter regulation (URAT1, ABCG2) and inflammatory signaling pathways and cytoprotective effects. However, findings obtained from these cellular systems should be considered mechanistic evidence and require validation in animal models and human studies. Caco-2 cells have been used directly to examine intestinal uric acid transport and ABCG2 function, while a recent HK-2 hyperuricemia model examined ROS and several renal urate transporters.

### 5.3. Animal Models and Translational Challenges

#### 5.3.1. Hyperuricemia Induction Models

The experimenta induction of hyperuricemia in animal models is a fundamental step in evaluating the preclinical effects of probiotic interventions and elucidating the mechanisms underlying uric acid dysregulation. Because most laboratory animals, particularly rodents, express uricase, which converts uric acid into allantoin, they are relatively resistant to sustained hyperuricemia compared to humans. Therefore, specific induction strategies are required to model elevated uric acid levels, where uricase is absent in humans [78]. These strategies are generally categorized into pharmacological, dietary, and combined approaches, each reflecting different aspects of disease pathophysiology [78]. Importantly, no single animal model fully reproduces the multifactorial nature of human hyperuricemia.

#### 5.3.2. Pharmacological Models: Potassium Oxonate-Induced Hyperuricemia

The pharmacological inhibition of uricase using potassium oxonate is acommonly employed method for inducing hyperuricemia. Potassium oxonate blocks uricase activity, thereby preventing the conversion of uric acid into allantoin and leading to its accumulation in the circulation [78]. This model is characterized by its rapid onset and relative experimental reproducibility, making it particularly suitable for short-term experimental studies. From a mechanistic standpoint, this model primarily reflects impaired uric acid degradation rather than increased production or altered uric acid excretion [78]. Consequently, it provides a simplified representation of hyperuricemia, focusing on a single metabolic pathway. Despite its utility, the model has notable limitations, including its inability to mimic the multifactorial nature of human hyperuricemia and its lack of interaction with dietary and microbiota-related factors. In addition, it typically represents an acute condition, which differs from the chronic progression observed in humans [78].

#### 5.3.3. Dietary Models: Yeast Extract and High-Purine Diets

Dietary induction models rely on the administration of purine-rich diets, commonly incorporating yeast extract or nucleic acid-rich components. Yeast extract is particularly effective because of its high purine content, which increases substrate availability for xanthine oxidase and promotes uric acid production [78]. Unlike pharmacological models, dietary approaches can model the contribution of increased dietary purine exposure to hyperuricemia, where excessive purine intake contributes to disease development. However, they should not be considered complete representations of human lifestyle-related hyperuricemia, which is influenced by multiple dietary, metabolic, genetic, and renal factors. An important advantage of dietary models is the ability to incorporate diet–microbiota interactions that may be relevant when evaluating probiotic interventions. High-purine substrates can influence intestinal substrate availability and microbial metabolism, although the resulting microbiota changes depend on the specific diet and experimental design. However, dietary models generally exhibit a slower onset and greater variability, as outcomes depend on factors such as diet composition, duration, and individual animal responses. Additionally, control over serum uric acid levels is less precise compared to pharmacological methods [78].

#### 5.3.4. Combined Models: Pharmacological and Dietary Approaches

To achieve a more comprehensive representation of hyperuricemia, many studies have employed combined models that integrate potassium oxonate administration with high-purine diets. This approach simultaneously targets multiple aspects of uric acid metabolism, including inhibition of degradation and increased production while also incorporating dietary and microbiota-related factors [78]. Combined models may provide a broader experimental representation of the complex interplay between metabolic, dietary, and microbial factors observed in human hyperuricemia. They also provide a useful platform for evaluating probiotics, which may influence relevant processes through multiple pathways. Nevertheless, these models introduce additional complexity, including increased variability and challenges in standardization [78].

#### 5.3.5. Advanced Models: Genetic and Humanized Systems

Recent advances have led to the development of genetic models, such as uricase knockout animals, which model the absence of uricase activity characteristic of humans. These models allow for sustained hyperuricemia without the need for chemical induction, providing a more sustained hyperuricemia model [78]. In parallel, integration with humanized microbiota systems can improve microbiome-related translational relevance. By incorporating human-derived gut microbiota, these models enable the study of microbiome-dependent mechanisms, which are particularly important in probiotic research [79]. Despite their advantages, both genetic and humanized models remain technically demanding and are not yet widely accessible [79].

#### 5.3.6. Evaluation Parameters in Hyperuricemia Models

The successful establishment of hyperuricemia induction and subsequent intervention effects was assessed through a range of biochemical, molecular, and histological parameters. Serum uric acid levels serve as the primary indicator, complemented by measurements of renal function markers such as creatinine and blood urea nitrogen [79,80]. The enzymatic activity of xanthine oxidase is often evaluated to assess metabolic changes, while inflammatory markers, including interleukin-1β and tumor necrosis factor-alpha, provide insights into immune responses. Oxidative stress was assessed using indicators such as malondialdehyde and antioxidant enzyme activity. Histopathological examination of renal tissues further reveals structural alterations and tissue damage [79,80].

#### 5.3.7. Relevance to Probiotic Research

Hyperuricemia models are particularly valuable for investigating the mechanisms of probiotic action. They enable the study of microbiota modulation, potential effects on intestinal uric acid excretion, renal transporter regulation, inflammation, and oxidative stress [78,80]. Dietary and combined models are especially relevant in this context, as they incorporate microbiota-related dynamics that are central to probiotic function [78,81]. Nevertheless, findings from these models should be interpreted as preclinical evidence.

#### 5.3.8. Critical Perspective: Translational Limitations

Despite their widespread use, hyperuricemia induction models have inherent limitations that must be carefully considered. The presence of uricase in most animal species represents a fundamental difference from human physiology, which may influence both disease progression and intervention outcomes [78]. Additionally, many models reflect acute hyperuricemia, whereas human hyperuricemia often develops over a prolonged period and is associated with complex metabolic disturbances. Interspecies differences in gut microbiota composition, immune responses, and metabolic regulation further limit direct translation [78,82]. Moreover, experimental conditions often fail to capture the complexity of human lifestyles, including dietary habits, comorbidities, and environmental influences. Consequently, findings from these models should be interpreted with caution and validated through well-designed clinical studies [78].

#### 5.3.9. Humanized Microbiota Models

Humanized microbiota models represent an important approach in translational research, designed to bridge the gap between preclinical findings and human physiological relevance in the gut.. These models involve the transplantation of human-derived gut microbiota into germ-free or microbiota-depleted animals, typically mice, thereby creating a system that partially reproduces the features of the human intestinal ecosystem [83]. In the context of hyperuricemia, such models provide a valuable platform for evaluating probiotic interventions under conditions that approximate the complexity and functionality of the human microbiome [32,83].

The establishment of humanized models generally begins with the use of germ-free animals, which are completely devoid of microorganisms, or animals treated with broad-spectrum antibiotics to significantly reduce the endogenous microbiota. These animals are then colonized by fecal microbiota obtained from human donors [83]. This process results in the formation of a “humanized” gut microbiome that retains aspects of the compositional and functional characteristics of the donor microbiota. One of the major advantages of humanized microbiota models is their ability to preserve aspects of donor-specific microbial signatures, including taxonomic composition, metabolic activity, and functional gene expression profiles [83]. This allows researchers to investigate how probiotic interventions interact with distinct human microbiome configurations, which is particularly important given the high interindividual variability observed in the gut microbiota [83].

In hyperuricemia research, these models may facilitate the study of microbiota-dependent mechanisms that cannot be fully captured in conventional animal models. For example, the impact of probiotics on purine metabolism, uric acid production, and microbial gene expression may be investigated in a system where the microbiota more closely resembles that of human patients [32,83]. This includes the evaluation of changes in genes involved in purine degradation, short-chain fatty acid synthesis, and urate transport modulation [32]. Furthermore, humanized models provide an opportunity to examine the interaction between probiotics and host microbiota crosstalk. Probiotic strains may behave differently depending on the existing microbial ecosystem, influencing colonization efficiency, metabolite production, and functional outcomes [32,83]. In this context, humanized models may help identify microbiota-dependent responses and potential responder versus non-responder phenotypes.

Another important application is the study of metabolic outputs and systemic effects. Humanized microbiota models can be used to assess changes in circulating metabolites, inflammatory markers, and renal function parameters following probiotic intervention [83]. This is particularly relevant for hyperuricemia, where systemic factors such as inflammation, oxidative stress, and kidney function play critical roles [32]. In addition, these models can be used to investigate fecal microbiota transplantation (FMT)-derived systems. Human-derived microbiota can be transferred into germ-free animals to examine microbiota-associated phenotypes [64]. This approach may help investigate the contribution of dysbiosis to hyperuricemia-related phenotypes [83]. Probiotic interventions can then be tested to determine their ability to reverse or modulate these disease-associated microbial profiles. Despite their advantages, humanized microbiota models have several important limitations. One major challenge is the incomplete replication of the human gut environment. Differences in host physiology, immune responses, diet, and intestinal anatomy can influence microbial colonization and function [83]. As a result, not all human microbes successfully engraft or maintain their original activity in animal hosts. Additionally, donor variability introduces significant heterogeneity in experimental outcomes. Microbiota from different individuals may respond differently to the same probiotic intervention, complicating data interpretation [83]. This variability, while reflective of real-world conditions, necessitates larger sample sizes and careful experimental design. Another limitation is the stability of the transplanted microbiota. Over time, the microbial community may shift due to adaptation to the host environment, leading to divergence from the original human profile. This can affect reproducibility and long-term studies [83].

## 6. Clinical Evidence and Translational Outlook

### 6.1. Overview of Clinical Trials

Clinical trials investigating the effects of probiotics on serum uric acid and hyperuricemia-related outcomes vary considerably in design, population characteristics, and intervention protocols. A recent meta-analysis identified nine eligible human studies involving 394 participants, including eight randomized controlled trials and one non-randomized comparative study, and reported substantial heterogeneity among the included studies [36]. There are vast differences in the probiotic formulations used in these studies, ranging from single-strain to multistrain formulations. Strains, doses, intervention periods, and participant characteristics differ substantially among studies [36]. This heterogeneity should be considered when comparing clinical outcomes. Serum uric acid has been a major outcome in these studies. The recent meta-analysis reported an overall significant reduction in uric acid with probiotics compared with control; however, substantial between-study heterogeneity was present, and individual studies did not show uniformly significant effects [36]. For example, a randomized double-blind controlled study involving 120 participants evaluated probiotic yogurt containing *Limosilactobacillus fermentum* GR-3 for two months. Serum uric acid decreased by 26.2% ± 2.3% in the probiotic-yogurt group compared with 8.6% ± 1.1% in the conventional-yogurt group [61,84]. In another randomized double-blind placebo-controlled study, 160 patients with gout received either Probio-X (3 × 10^10^ CFU/day) plus febuxostat or placebo plus febuxostat for two months. Probiotic coadministration was associated with lower serum uric acid and fewer acute gout attacks; importantly, this study evaluated probiotics as an adjunct to established urate-lowering therapy in patients with gout rather than as a stand-alone treatment for hyperuricemia [60]. Some trials have also reported changes in inflammatory, metabolic, microbiome, and metabolomic outcomes; however, these secondary effects vary according to the population, strain, and study design and should not be generalized across probiotic interventions [60,61,84].

In addition to metabolic outcomes, renal outcomes have been less consistently evaluated, and the available clinical evidence is insufficient to establish the nephroprotective effect of probiotics in hyperuricemia. Emerging clinical evidence also highlights the role of probiotics in modulating gut microbiota composition. Changes in microbial diversity and the relative abundance of specific taxa have been reported in selected studies, supporting further investigation of microbiota-mediated mechanisms [60,61,84]. Nevertheless, the integration of microbiome analysis into clinical trials remains limited, and functional insights are often lacking. The principal human studies discussed in this section are summarized in Table 3, including study population, probiotic formulation, dose, intervention duration, sample size, primary outcome, and reported effect on serum uric acid.

### 6.2. Limitations of Current Clinical Evidence

Despite encouraging findings, the current body of clinical evidence is subject to several important limitations that restrict its interpretability and generalizability. One of the most significant challenges is the small sample size of many available studies, which may limit statistical power and contribute to inconsistent results [36]. This limitation also reduces the ability to perform subgroup analyses, which are essential for understanding variability in treatment response. Another major limitation is the short duration of interventions. Many available studies are relatively short-term, which may be insufficient to capture long-term effects on uric acid metabolism and related complications. Hyperuricemia is a chronic condition, and short-term interventions may not reflect sustained effects [36]. Design heterogeneity further complicates the interpretation of clinical findings. Variations in probiotic strains, dosages, delivery formats, and co-interventions make it difficult to compare results across studies. In addition, differences in participant characteristics, including baseline uric acid levels, dietary habits, and comorbidities, contribute to variability in outcomes [36].

A particularly critical limitation is the lack of mechanistic studies in human trials. While preclinical research has identified multiple pathways through which probiotics may influence uric acid metabolism, these mechanisms are rarely validated in clinical settings. Most trials focus on clinical endpoints without integrating molecular or microbiome analyses, resulting in a gap between observed effects and underlying biological processes [83]. Moreover, adherence to probiotic supplementation and lifestyle factors, such as diet, are not always adequately controlled or reported, introducing potential confounding variables. The placebo effect and variations in gut microbiota composition among individuals further add to the complexity of interpreting results [36].

### 6.3. Future Directions for Clinical Research

To advance this field, future clinical research should adopt more rigorous and integrative approaches that address current limitations and enhance translational relevance. One promising direction is the development of personalized probiotic interventions based on baseline microbiota profiles. Given the substantial interindividual variability in gut microbiota composition, tailoring probiotic selection to the specific microbial landscape of each individual may profiles improve response and reduce variability in treatment outcomes. This approach aligns with the emerging concept of precision medicine. In parallel, the incorporation of multi-omics technologies, including metabolomics and metagenomics, can provide deeper insights into the mechanisms underlying probiotic action in humans. Biomarker-guided monitoring allows the identification of functional changes in metabolic pathways, microbial gene expression, and host responses. This integrative strategy can help bridge the gap between clinical observations and mechanistic understanding [44]. Future studies should also prioritize larger sample sizes and longer intervention periods to improve statistical power and capture long-term effects. The standardization of probiotic formulations, including strain selection, dosage, and delivery methods, is essential for ensuring reproducibility and comparability across studies [36]. Additionally, well-designed randomized controlled trials should incorporate comprehensive endpoint assessments, including metabolic, inflammatory, renal, and microbiome-related parameters. This holistic evaluation is necessary to better characterize the potential effects of probiotics in hyperuricemia [36,47].

## 7. Challenges and Future Perspectives

Despite the growing body of evidence investigating probiotics in relation to hyperuricemia, several scientific, technological, and regulatory challenges must be addressed to facilitate their translation into effective clinical and functional food applications. Emerging, emerging innovations offer promising opportunities to improve probiotic functionality and precision [36,44].

### 7.1. Strain Viability and Delivery Optimization

One of the primary challenges in probiotic applications is maintaining strain viability and functional activity throughout processing, storage, and gastrointestinal transit. Probiotic microorganisms are highly sensitive to environmental stressors, including temperature fluctuations, oxygen exposure, and gastric acidity, all of which can significantly reduce their survival and functional performance [10,44]. Advanced encapsulation technologies have been developed to overcome these limitations.. Microencapsulation techniques involve embedding probiotic cells within protective matrices, such as alginate or chitosan, which shield them from harsh conditions during storage and digestion. More recently, nanocarrier-based delivery systems have emerged, offering enhanced protection, controlled release, and improved stability. These nanoscale systems can be engineered to respond to specific physiological triggers, such as pH or enzymatic activity [10,44].

In addition to protection, targeted delivery systems are being investigated to improve probiotic delivery. Colon-targeted delivery approaches are particularly relevant, as many probiotic functions, including SCFA production and microbiota modulation, occur primarily in the large intestine. Strategies such as pH-sensitive coatings and time-dependent release formulations are designed to facilitate passage through the upper gastrointestinal tract and reach their site of action more efficiently. Despite these advancements, challenges remain in balancing technological complexity with cost-effectiveness and scalability, which are essential for widespread clinical and commercial applications.

### 7.2. Regulatory Frameworks and Health Claims

The regulatory landscape for probiotics and functional foods varies significantly across countries, presenting significant challenges for product development and global commercialization. The regulatory classification and requirements for probiotic products differ according to jurisdiction and the intended product category, including food, dietary supplements, and live biotherapeutic products [47]. However, the approval of health claims associated with probiotic products requires robust scientific evidence. Regulatory agencies typically demand well-designed clinical trials that demonstrate clear and reproducible health benefits [47]. Another challengeis the strain-specific nature of probiotic effects. Health claims must be supported by evidence of the exact strain used, rather than being generalized at the species level [47]. This requirement necessitates the extensive characterization and validation of individual strains, thereby increasing the complexity and cost of development. Moreover, inconsistencies in labeling, dosage recommendations, and quality control standards can undermine consumer confidence and limit their clinical adoption. Therefore, harmonizing of regulatory frameworks and establishing standardized guidelines are critical for advancing the field [10].

### 7.3. Emerging Technologies and Next-Generation Probiotics

**Figure 5:**
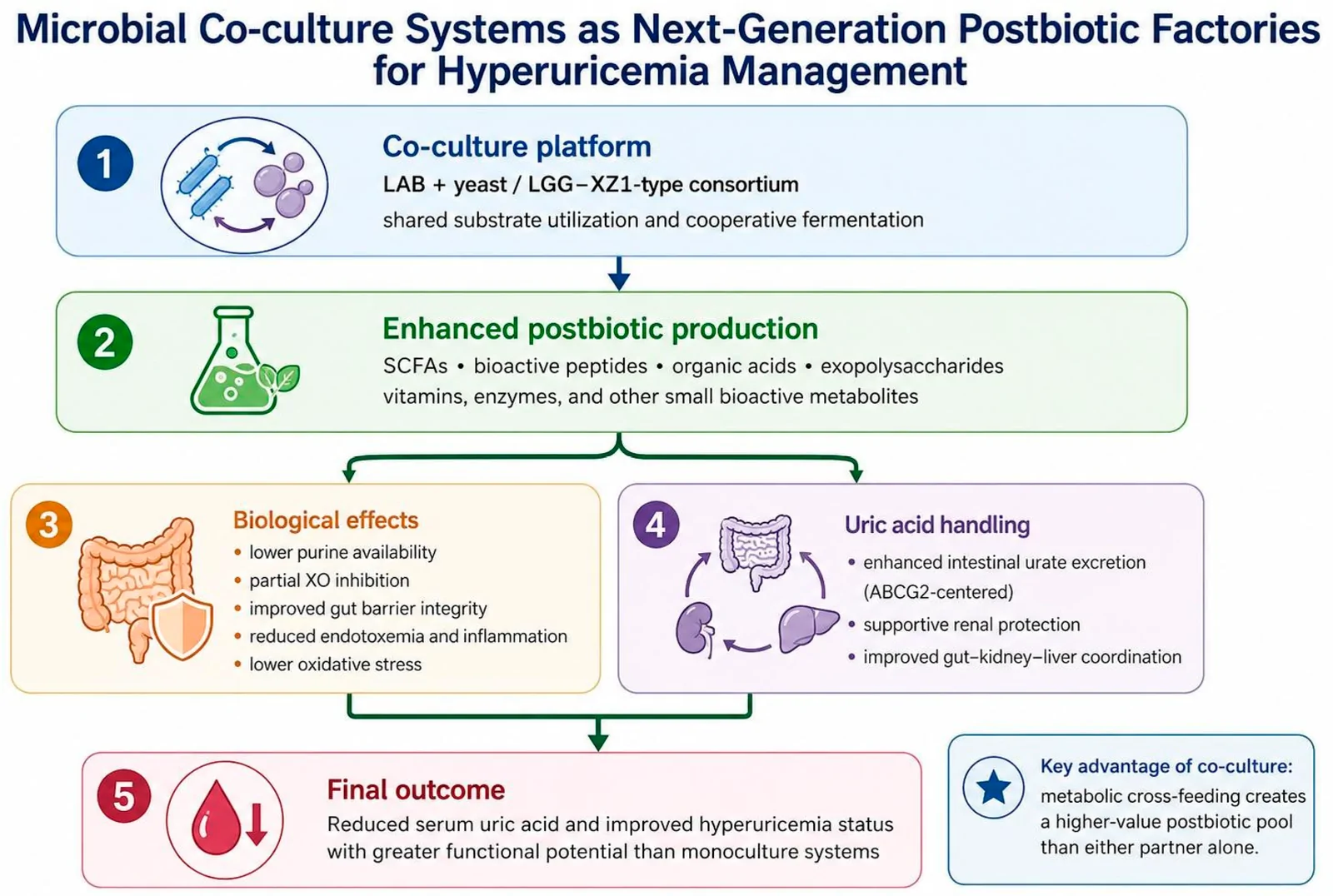
Conceptual summary of microbial co-culture systems as postbiotic factories for hyperuricemia management. The numbered panels indicate the proposed sequence from (1) a LAB–yeast co-culture platform, (2) generation of fermentation-derived bioactive products, (3) potential biological effects related to purine metabolism, intestinal barrier function, inflammation, and oxidative stress, (4) possible modulation of intestinal and renal urate-handling pathways, to (5) a potential reduction in serum uric acid. Blue denotes the co-culture platform, green denotes fermentation-derived bioactive production, orange denotes proposed biological effects, purple denotes uric acid-handling pathways, and red denotes the potential final outcome. The microbial icons represent bacterial and yeast partners; the flask represents fermentation-derived products; the intestine/shield icon represents gut-related biological effects; the gut–kidney–liver schematic represents urate-handling pathways; and the blood-drop/downward-arrow icon represents a potential decrease in serum uric acid. Arrows indicate proposed mechanistic or functional relationships rather than established causal effects. The star symbol highlights the hypothesized advantage of co-culture systems through complementary metabolic interactions.

Recent advances in biotechnology and computational science have driven the development of next-generation probiotics with enhanced functionality and precision. One of the most promising approaches involves the use of synthetic biology to engineer probiotic strains with targeted metabolic capabilities. These approaches have the potential to improve probiotic functionality by modifying specific microbial traits; however, their application in hyperuricemia remains largely conceptual and requires extensive validation regarding safety, efficacy, and regulatory acceptance [10,35]. The application of CRISPR-Cas systems provides a powerful tool for precise genetic modification of probiotic strains. This technology enables the insertion, deletion, or regulation of specific genes involved in purine metabolism, antioxidant defense, and inflammatory pathways. Although CRISPR-based approaches provide opportunities for targeted strain development, genetically modified probiotics require careful assessment of biological stability, biosafety, and regulatory requirements before clinical translation [47]. In parallel, artificial intelligence and machine learning are increasingly being used to optimize probiotic selection and formulation. By integrating multi-omics data, including metagenomics, metabolomics, and transcriptomics, AI-driven models can identify optimal strain combinations and predict their functional interactions within the host environment. Nevertheless, the use of AI-based approaches in probiotic development remains an emerging field, and predictive models require validation using experimental and clinical datasets [47,85]. This approach supports the development of personalized probiotic therapies tailored to individual microbiota profiles. Despite their potential, these advanced technologies raise important ethical, regulatory, and safety considerations. The use of genetically engineered microorganisms, in particular, requires rigorous evaluation to ensure long-term safety and public acceptance. Therefore, these technologies should be considered enabling platforms for future probiotic development rather than established therapeutic strategies for hyperuricemia. Microbial co-culture systems were chosen for this review, and, specific to this review, an integrative summary figure was prepared to visually synthesize the potential relationship between probiotic–postbiotic synergy and microbial co-culture strategies [66] (Figure 5) summarizes the proposed relationship between microbial co-culture systems, postbiotic production, and potential anti-hyperuricemic mechanisms discussed throughout this review [36,81].

## 8. Conclusions

Probiotic-based strategies for hyperuricemia management have evolved from simple single-strain interventions to complex, multitarget approaches that address the multifactorial nature of the disease. Accumulating evidence demonstrates that probiotics can modulate uric acid metabolism through a wide range of mechanisms, including inhibition of purine degradation pathways, enhancement of intestinal and renal uric acid excretion, regulation of gut microbiota composition, and attenuation of inflammation and oxidative stress. Recent advances have highlighted the potential value of combining probiotics with complementary functional components, including prebiotics, postbiotics, polyphenols, and bioactive peptides. However, the clinical superiority of these combined strategies over single-strain approaches remains to be established through well-designed comparative human studies [36,59,60]. These integrated strategies provide broader and more effective modulation of metabolic and physiological pathways compared to single-strain approaches. Multistrain formulations further enhance this effect by combining complementary functions within a single system [61]. Importantly, microbial co-culture systems represent a promising strategy for enhancing microbial cooperation and postbiotic production, providing a potential translational bridge between mechanistic understanding and functional product development. Nevertheless, further studies are required to define optimal microbial combinations, reproducibility, and clinical relevance. Despite promising preclinical and early clinical findings, significant challenges remain in translating these strategies into clinical practice [36]. Variability in probiotic strains, limited standardization, and insufficient mechanistic validation in human studies continue to hinder progress. Addressing these limitations requires well-designed clinical trials, standardized methodologies, and integration of advanced analytical techniques. Future research may benefit from precision nutrition approaches in which probiotic interventions are tailored according to individual microbiota profiles and metabolic characteristics; however, clinical implementation requires reliable biomarkers and validation in diverse human populations [62].

## Figures and Tables

**Table 1:** Major gut microbial taxa and their functional roles in the regulation of purine metabolism.

Genus/Species	Reported Metabolic Effect in the Cited Study	Primary Metabolic Function	Key Genes/Enzyme	Impact on Uric Acid Levels	Evidence Type	Experimental System/*n*
*Klebsiella pneumoniae *(strain not specified in the primary study)	Increased plasma UA in the cited germ-free mouse model	Associated with microbial purine degradation pathways	hpxQ association reported in the metagenomic analysis	Significant increase in plasma UA; the exact fold-change was not stated in the primary text	Germ-free mouse experiment + multi-omics	10-week-old germ-free male C57BL/6J mice; *n* = 5/group
*Enterococcus faecalis *(strain not specified in the cited intervention study)	Increased serum UA in the specific inoculation model	XOD-producing bacterium as defined in the cited mouse model	Not directly measured in the intervention study	↑ Serum uric acid (significant increase compared with the blank group)	Mouse intervention model	Male C57BL/6J mice; six experimental groups; *n*/group should be reported from the full primary dataset if available
*Lactobacillus reuteri TSR332*	Reduced serum UA in the cited hyperuricemic rat model	Purine-nucleoside assimilation (inosine and guanosine)	These enzymes were not directly quantified as the principal endpoint in the cited study	Serum UA ↓60%; inosine assimilation 90%; guanosine assimilation 78%	In vitro screening + hyperuricemic rat model	Wistar rats; *n* = 8/group
*Bifidobacterium longum* (strain must be specified in the primary study)	Reported potential urate-lowering effect; interpretation must remain strain-specific	The original mechanism requires confirmation from the primary study	Primary experimental confirmation required	Quantitative SUA effect should be reported only after confirmation from the primary source	Evidence type to be specified from the primary source	To be completed from primary study
*Faecalibacterium prausnitzii *(strain not specified)	Associated with gout-related microbial and butyrate-metabolism differences; direct urate-lowering action not established	Butyrate-producing taxon depleted in gout-associated microbiota	A causal urate-lowering role for these genes was not established in the cited human study	No direct 28% SUA or 30% hepatic XDH reduction should be retained unless the primary experimental source for these values is identified	Human 16S rRNA/metagenomic association study	35 gout patients and 33 healthy controls; additional validation cohort *n* = 15
*Clostridium difficile *(strain not specified)	Reported purine-metabolism association in the current secondary source	Increased purine degradation activity	xdhC, purF	Direct quantitative effect on SUA requires confirmation from the primary study	Metagenomic analysis	Primary experimental system/n must be identified
*Escherichia coli *(strain not specified in the host-UA experiment)	Increased plasma UA in the cited germ-free mouse model	Carries XDH-like gene clusters that enhance purine oxidation	xdhA/B/C	Significant increase in plasma UA; the 2.3-fold value should not be retained unless confirmed from the primary dataset	Multi-omics + germ-free mouse experiment	10-week-old germ-free male C57BL/6J mice; *n* = 5/group

**Table 2:** Comparative overview of major probiotic-based strategies for hyperuricemia management.

Strategy	Main Mechanism(s)	Level of Evidence	Advantages	Limitations
Probiotics	Microbiota modulation; strain-specific purine metabolism; potential XO-related effects; barrier support	Predominantly preclinical; limited and heterogeneous clinical evidence	Living, dynamic, potentially multitarget	Viability and context-specific effects
Synbiotics	Live microorganisms combined with substrate(s) selectively utilized by host microorganisms; potential support of microbial activity and SCFA production	Mainly preclinical; limited clinical evidence	Potential support for microbial persistence and metabolic activity	Formulation complexity and substrate dependence
Postbiotics	Preparations of inanimate microorganisms and/or their components with potential biological activity	Mainly preclinical; limited hyperuricemia clinical evidence	Improved stability and opportunities for standardization; no requirement for microbial viability	Preparation-specific effects; limited hyperuricemia clinical evidence
Polyphenol + Probiotics	Potential complementary antioxidant, anti-inflammatory, XO-related, and microbiota effects	Predominantly preclinical	Potential dual host–microbe functionality	Matrix and dose dependence; direct combination evidence in hyperuricemia remains limited
Peptide + Probiotics	Potential complementary XO-related, antioxidant, inflammatory, and barrier effects	Emerging preclinical	Potential complementary bioactivities	Peptide stability; limited direct combination and clinical evidence
Herbal Extract + Probiotics/Fermentation (NEW ROW)	Microbial biotransformation of phytochemicals; potential XO-, microbiota-, inflammatory-, and urate-transporter-related effects	Direct preclinical evidence for selected fermented herbal preparations; limited human evidence; emerging preclinical/process development stage	Potential enhancement of phytochemical transformation and functional food development	Strong strain-, herb-, fermentation-, and formulation-dependence; limited clinical validation
Multistrain Probiotics (NEW ROW)	Functional complementarity, metabolic cross-feeding, and overlapping microbial functions	Mainly mechanistic and preclinical; limited direct hyperuricemia evidence	Potentially broader functional coverage and resilience	Strain compatibility, dose ratios, host dependence, and uncertain superiority over single strains
Co-culture Systems	Metabolic cross-feeding and generation of broader fermentation-derived product profiles; potential postbiotic development	Emerging preclinical and process-development evidence; direct human evidence lacking	Potentially broader metabolite profile; cross-feeding; tunable design	Process optimization, strain compatibility, batch variability, biosafety assessment, and regulatory uncertainty

**Table 3:** Human clinical studies evaluating the effects of probiotic and probiotic–postbiotic interventions on serum uric acid and gout-related outcomes.

Study	Population/Design	Intervention and Dose	Duration	Sample Size	Main Outcome	Interpretation
[36]	Adults with marginal hyperuricemia; randomized, double-blind, placebo-controlled	*Lactobacillus gasseri* PA-3 yogurt; low 3 × 10^6^ CFU/g and high 3 × 10^7^ CFU/g	8 weeks	60	Overall, SUA difference was not significant; dietary purine-adjusted analysis showed lower SUA with high-dose PA-3	Mixed evidence
[37]	Healthy adults; randomized crossover purine challenge study	*L. gasseri* PA-3 yogurt (112 g/day) with purine nucleotide challenge	4 days	16	Reduced postprandial SUA increase and lower SUA AUC	Mechanistic human evidence
[85]	Hyperuricemia/gout patients; randomized double-blind placebo-controlled	*L. gasseri* PA-3 yogurt ≥8.5 × 10^7^ CFU/g, 100 g/day	8 weeks	25 completed	No significant overall SUA reduction; subgroup effect observed	Mixed/limited evidence
[84]	Adults with hyperuricemia; randomized controlled trial	*Limosilactobacillus fermentum* GR-3 yogurt, 250 g/day	2 months	120	SUA decreased 26.2% vs. 8.6% control	Direct positive HUA evidence
[85]	Hyperuricemia with recurrent gout; randomized pilot trial	*Ligilactobacillus salivarius* CECT 30632 (~10^9^ CFU/day)	6 months	30	SUA decreased from 9.04 to 7.90 mg/dL	Positive pilot evidence
[84]	Adults with metabolic risk; randomized placebo-controlled trial	Multistrain probiotics ± PE0401 postbiotic; 2.01 × 10^10^ CFU/day	60 days	82	3Mix + postbiotic showed greatest SUA reduction, especially in males	Relevant probiotic–postbiotic evidence
[85]	Gout patients receiving febuxostat; randomized controlled adjunctive trial	Probio-X mixture, 3 × 10^10^ CFU/day + febuxostat	2 months	160	Improved SUA response and reduced gout attacks	Adjunctive evidence
[77]	Hyperuricemia patients; randomized double-blind trial	*Lactobacillus rhamnosus* CCFM1131, 6 × 10^9^ CFU/day	8 weeks	107	Reduced SUA and XOD activity	New direct HUA evidence
[13]	Metabolic syndrome; randomized trial	*L. acidophilus* La5 + *B. animalis* Bb12 yogurt	8 weeks	44	SUA decreased in probiotic yogurt group	Supportive evidence
[60]	Hemodialysis patients; randomized trial	Multistrain probiotic powder	12 weeks	75	No meaningful SUA reduction	Negative evidence
[22]	Healthy adults; placebo-controlled trial	*Bifidobacterium* animalis BLa80, 10^10^ CFU/day	8 weeks	No significant SUA lowering	Negative evidence	

## Data Availability

The original contributions presented in this study are included in the article. Further inquiries can be directed to the corresponding author.

## Abbreviations

The following abbreviations are used in this manuscript:

ABC	ATP-binding cassette
ABCC2	ATP-binding cassette subfamily C member 2
ABCG2	ATP-binding cassette subfamily G member 2
ADA	Adenosine deaminase
AhR	Aryl hydrocarbon receptor
AI	Artificial intelligence
AMPK	AMP-activated protein kinase
Caco-2	Human colorectal adenocarcinoma cell line
CRISPR-Cas	Clustered regularly interspaced short palindromic repeats–CRISPR-associated protein system
FMT	Fecal microbiota transplantation
FOSs	Fructooligosaccharides
GLUT9	Glucose transporter 9
GMP	Guanosine monophosphate
GOSs	Galactooligosaccharides
HK-2	Human kidney-2 proximal tubular epithelial cell line
IL-1β	Interleukin-1 beta
IL-6	Interleukin-6
IL-10	Interleukin-10
LAB	Lactic acid bacteria
LGG	Lacticaseibacillus rhamnosus GG
LPS	Lipopolysaccharide
NADPH	Reduced nicotinamide adenine dinucleotide phosphate
NF-κB	Nuclear factor kappa B
NLRP3	NOD-like receptor family pyrin domain-containing 3
Nrf2	Nuclear factor erythroid 2-related factor 2
PNP	Purine nucleoside phosphorylase
PPARs	Peroxisome proliferator-activated receptors
ROS	Reactive oxygen species
SCFAs	Short-chain fatty acids
SUA	Serum uric acid
TLR4	Toll-like receptor 4
TNF-α	Tumor necrosis factor-alpha
URAT1	Urate transporter 1
XDH	Xanthine dehydrogenase
XO/XOD	Xanthine oxidase
ZO-1	Zonula occludens-1
